# *‘Mankind owes to the child the best that it has to give’*: prison conditions and the health situation and rights of children incarcerated with their mothers in sub-Saharan African prisons

**DOI:** 10.1186/s12914-019-0194-6

**Published:** 2019-03-05

**Authors:** Marie-Claire Van Hout, Rosemary Mhlanga-Gunda

**Affiliations:** 1 Public Health Institute, Liverpool John Moore’s University, Liverpool, L32ET UK; 2 College of Health Sciences, Centre for Evaluation of Public Health Interventions, Department of Community Medicine, University of Zimbabwe, Harare, Zimbabwe; 3 Public Health Institute, Exchange Station, Liverpool John Moore’s University, Liverpool, L32ET UK

**Keywords:** Sub Saharan Africa, Prisons, Women, Infants, Children, availability and accessibility of health services, availability of basic necessities, human immunodeficiency virus infection, (HIV)

## Abstract

**Background:**

In recent times, sub-Saharan African (SSA) prisons have seen a substantial increase in women prisoners, including those incarcerated with children.

**Methods:**

A scoping review mapped what is currently known about the health situation and unique rights violations of children incarcerated with their mothers in SSA prisons. A systematic search collected and reviewed all available and relevant published and grey literature (2000–2018). Following application of exclusion measures, 64 records remained, which represented 27 of the 49 SSA countries. These records were charted and thematically analysed.

**Results:**

Four main themes were generated as follows: 1) the prison physical environment; 2) food availability, adequacy and quality; 3) provision of basic necessities and 4) availability and accessibility of health services for incarcerated children.

**Conclusions:**

The review highlights the grave situation of children incarcerated with their mothers in SSA prisons, underpinned by the lack of basic necessities, inadequate hygiene, sanitation and safe drinking water, exposure to diseases in overcrowded cells, inadequate nutrition, lack of provision of clothing and bedding, and difficulties accessing paediatric care. Reported paediatric morbidity and mortality associated with such prison conditions is deeply concerning and contrary to international mandates for the rights of the child, right to health and standards of care.

**Electronic supplementary material:**

The online version of this article (10.1186/s12914-019-0194-6) contains supplementary material, which is available to authorized users.

## Background

Approximately 6.5% of the world’s prisoners are women [1]. Whilst a minority, more than 500,000 women and girls are held in prisons and other closed settings, both as sentenced prisoners or as pre-trial detainees [2]. This number has increased by about 50% since 2000 in comparison to an 18% increase in the male population, and is rising in all regions of the world where statistics are available [1]. The dramatic increase in imprisoned women is important from a public health perspective. Women’s special health needs relating to specific health approaches, sexual and reproductive health (SRH) care needs, the treatment of infectious diseases, nutrition and female hygiene requirements are often neglected in prisons and other closed settings [3, 4]. Incarcerated women generally experience gender-specific health-related challenges, which include menstruation, pregnancy and childbirth, care of their children within and outside of prison, development of certain forms of cancer, and are often exposed to gender-based violence in the form of physical/sexual abuse by prison officers and male prisoners [5–8]. Concerns around equitable quality and access to adequate health care for incarcerated women and their children are evident [9] .

Humane treatment of incarcerated women, and provision of adequate health services for women (and their infants and children) in prisons are mandated under the Sustainable Development Goals (SDG’s) 3, 5, and 16, as well as under United Nations instruments; Standard Minimum Rules for the Treatment of Prisoners (Nelson Mandela Rules) (A/RES/70/175) [10] Standard Rules for Non-Custodial Measures (Tokyo Rules) [11] and Rules for the Treatment of Women Prisoners and Non-Custodial Measures for Women Offenders (Bangkok Rules) (A/RES/65/229) [12] The Bangkok Rules in particular stipulate the standards for healthcare programming equivalent to that in the community and recognition of women’s specific health needs during incarceration, and also in relation to their children who reside in prisons with their mothers. Overarching these rules is the United Nations (UN) Convention on the Rights of the Child [13]. The 2010 UN Guidelines for the Alternative Care of Children mandate that: “best efforts should be made to ensure that children remaining in custody with their parent benefit from adequate care and protection, while guaranteeing their own status as free individuals and access to activities in the community” [14].

The sub-Saharan African (SSA) region continues to be the epicentre of the HIV epidemic, with two-thirds of all people infected with the human immunodeficiency virus (HIV) living in this region, and with high rates of HIV reported in prisons [15, 16]. Female sex is correlated with prevalent HIV infection in SSA prisons [15]. According to a recent evaluation by the United Nations Office on Drugs and Crime (UNODC), the overwhelming majority of prisoners in SSA, regardless of age and gender are detained under conditions that do not meet or only partially meet accepted standards of care [17]. Prison environments in the SSA region are compromised by weak prison and public health systems, failing prison infrastructure and ineffective criminal justice systems with high rates of pre-trial detention [18]. Investment in prison infrastructure is generally low across the SSA region [18]. In many SSA countries, pre-trial detainees can remain awaiting trial for lengthy periods (sometimes years) and this exacerbates the impact of such poor conditions of detention. As a consequence, overcrowding is pervasive. It is therefore not only imprisoned mothers with their children that are suffering such poor conditions, but the entire prison population in SSA [17, 18]. Weak prevention and treatment interventions for HIV, tuberculosis (TB), cholera, and malaria in prisons exacerbate the spread of disease [18, 19].

Children incarcerated with their mothers in the SSA region are a particularly under-researched and vulnerable group [5] often described as “hidden victims”, with “their reality and circumstances related to incarceration seldom recognised” [20]. The children of particular concern to policy-makers and researchers are those born in prison and those under the age of eight years [21–25]. Potential factors supporting the incarceration of children with their mothers include optimal duration of breastfeeding, strengthening of mother-to-child bonds in early development and the inability of the mother to arrange alternative care for her child [5, 26]. With regard to children incarcerated with their mothers in SSA, the African Charter on the Rights and Welfare of the Child (ACRWC) [27] affirms the principle of the best interests of the child, with Article (19) stating that “the child shall be entitled to the enjoyment of parental care and protection and shall, whenever possible, have the right to reside with his or her parents. No child shall be separated from his parents against his will, except when a judicial authority determines in accordance with the appropriate law that such separation is in the best interest of the child.” Of note however is that SSA prisons generally do not budget for the cost of looking after children born in prison and/or incarcerated with their mothers [18].

Research activity on prison populations and their health needs remains scant in the SSA region, and remains largely restricted to the gathering of strategic information on infectious diseases such as HIV and TB, and generally conducted in adult male prisons [15, 18]. Very little work has been done on women and their children incarcerated in the SSA region. A 2018 review has highlighted the abhorrent prison conditions for incarcerated women, and neglect of their specific health rights and needs in this region [18]. To date, there has not been an extensive review of published material on the conditions of children incarcerated with their mothers in SSA. The present review seeks to fill that gap.

## Methods

Scoping reviews are defined as a form of research synthesis that aims to map the literature on a particular topic or research area and are used to identify key concepts; gaps in research, and types and sources of evidence to inform practice, policymaking and research [28–31]. The scoping review process was conducted by two authors with relevant expertise in community medicine, prison and public health, gender and African health systems [28]. The underpinning research question was; “What is known in the literature about the health situation and rights violations specific to children incarcerated with their mothers in contemporary SSA prisons?” The term “prison” was adopted as representing facilities housing both on-remand female prisoners (including jails, police holding cells, and other closed settings) and convicted female prisoners [18]. We restricted the scoping exercise to all records reporting on the situation of children incarcerated with their mothers and including those born in prison and those below the age of eight years permitted by prison services in SSA to be housed with their mothers. The six-stage iterative process guiding the scoping review consisted of (1) identifying the research question, (2) identifying relevant studies, (3) study selection, (4) charting the data, (5) collating, summarizing and reporting the results, and (6) an international expert advisory review exercise [28] . Search terms were generated in English, and combined with SSA country names. The search strategy is illustrated in Table 1.

**Table 1:** ‘*Search Terms and Strategy’*

Key Word	Alternative
Children in Prisons	Circumstantial children in prisons, OR children accompanying their mothers in prison, OR children imprisoned with their mothers , OR children incarcerated with their mothers
Research evidence	AND availability and accessibility of healthcare OR availability of nutrition OR availability of basic necessities OR availability of HIV/AIDS treatment OR physical environment structure
African Countries	Sub Saharan Africa OR Africa OR and the names of all the individual countries in Sub Saharan Africa
1. Children in prisons 2. Circumstantial children in prisons OR children accompanying their mothers in prison OR children imprisoned with their mothers OR children incarcerated with their mothers 3. OR health services availability and accessibility, OR availability of basic necessities OR availability of nutrition, OR availability of HIV/IDS treatment, OR physical environment) AND 4 Africa Databases were searched using the appropriate subject headings and/or keywords or text words for the above search groups: Sample Search (Pubmed Central) searched on 29-03-2018 # Searches Results 1. Circumstantial children in prisons OR children accompanying their mothers in prisons OR Children imprisoned with their mothers OR children incarcerated with their mothers 2. Health services availability and accessibility OR availability of basic necessities OR availability of nutrition OR availability of HIV/IDS treatment, OR physical environment) AND Africa 197	

The search was implemented in April and May 2018 in the University of Zimbabwe and Liverpool John Moore’s University Library catalogues, PubMed Clinical Queries, and Scopus (exploratory search with selected references downloaded for the purpose of clarifying search terms). Comprehensive searches restricted to the time period 2000 to 2018 were conducted in the Cochrane Library, Science Direct, PubMed, EBSCO, Host, Embase, Medline, Embase, Medline in Process, PsycINFO and CINAHL.

To enable the broadest picture of current knowledge and perceptions relating to the issue of infants’ and childrens’ health in SSA prisons, we included international and national policy documents and reports, academic theses, online reports, country situational assessment reports conducted by national, international and human rights organisations, conference proceedings, commentary pieces and editorials, in addition to articles in scholarly peer- reviewed journals. We included reports by the Special Rapporteur on Prisons and Conditions of Detention in Africa (hereinafter Special Rapporteur) who assess whether conditions in prisons and other closed settings are compliant with the African Union (AU) Member States’ international obligations toward persons deprived of liberty. Where possible, we also included studies providing information about prison staff members’ experiences and perspectives on the conditions and rights of infants and children’ in SSA prisons. Follow-up search strategies included website searches of international aid, human rights and development organisations, health, medical and human rights-related databases, websites of SSA government and non-governmental organisational (NGO) bodies and investigative news reports. Reference lists in reports, investigative news articles, journal papers and academic theses were also manually searched by the team to identify any additional relevant literature not captured.

**Fig. 1:**
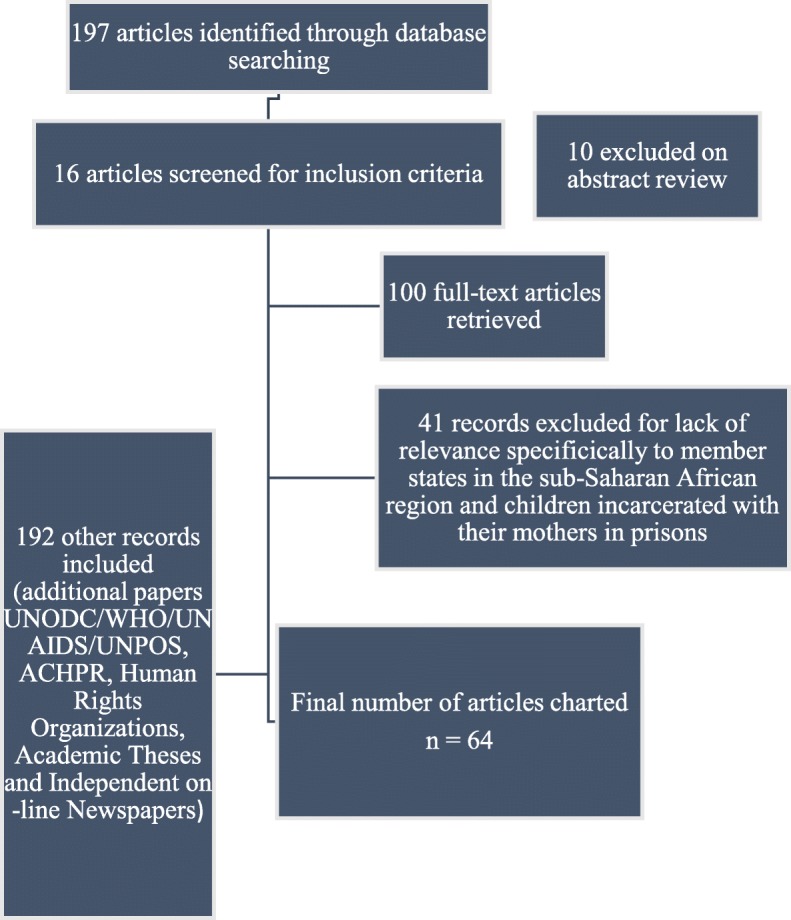
‘Flowchart for inclusion and exclusion of literature’

Records were managed using EndNote. The title and abstract of each record were screened by the second author, and cross-checked by the first author [28]. All records warranting inclusion were procured for review of the full text version. A second screen of the full text of each record was conducted by both authors. Studies were excluded at this stage if found not to meet the eligibility criteria. Figure 1 reflects inclusion and exclusion criteria used to chart the studies.

Following application of exclusion measures, 64 records were charted and thematically analysed, as per Levac et al. [28]. This process of documentation and analysis of information generated specific themes pertaining to incarcerated children and infant experiences, health outcomes and unique prison health care needs in the SSA region. A spreadsheet was created to chart relevant data (data collection categories, year of publication, author, location, method and aim, key findings and conclusion) and identify commonalities, themes, and gaps in the literature. We conducted a trial charting exercise of several records as recommended by Daudt et al. [30], followed by a joint consultation to ensure consistency with the research question and the purpose of the scoping review. Based on this preliminary exercise, we developed prior categories which guided the subsequent extraction and charting of the data from the records. All records were charted and analysed by the two reviewers in consultation, with disagreements around theme allocation resolved through discussion. Where additional data extraction categories emerged, consultation guided decisions around allocation and reporting. Identified themes were further presented and discussed with key experts from the SSA region [28] with expertise in prison health, health rights, SRH programming and international aid, to ensure no useful records were missed and to elicit varied perspectives on incarcerated children living with their mothers in SSA prisons.

## Results

Literature was found representing 27 of the 49 SSA countries. These were Benin, Botswana, Burundi, Cameroon, Chad, Côte d’Ivoire, Djibouti, Ethiopia, Ghana, Kenya, Madagascar, Malawi, Mali, Mozambique, Namibia, Nigeria, Rwanda, Senegal, Sierra Leone, Somalia, South Africa, South Sudan, Swaziland, Tanzania, Uganda, Zambia and Zimbabwe. We present the countries with corresponding type of record (for example journal paper, report, etc) in Table 2. For illustrative purposes where possible, we present quotes from included qualitative studies.

**Table 2:** ‘*Summary table of country records’*

		Number of results per category							
Country	Journal Articles	United Nations Reports	African Union Reports	Human Rights reports	Chapter in a book	Government reports/ Minutes	Academic Thesis	Independent on-line Newspapers	Total all categories per country
Benin						1			1
Botswana						1			1
Burundi						1			1
Cameroon	2		1						3
Chad				1					1
Côte d’Ivoire						2			2
Djibouti						1			1
Ethiopia			1			1			2
Ghana	1	1							2
Kenya	2					1	1		4
Madagascar						1			1
Malawi			1				1		2
Mali						1			1
Mozambique	1		1						2
Namibia			1			1			2
Nigeria	1					1			2
Rwanda		1							1
Senegal						1			1
Sierra Leone				1		1			2
Somalia		1							1
South Africa	1	1	1			1	1		5
Swaziland						1			1
Sudan		1				1			2
Tanzania						1			1
Uganda		1	1			1		1	4
Zambia	3			1		1	1	1	7
Zimbabwe	1				1	1		3	6
Total									59 *

*Five charted results not included in Table 2 are Agomoh (2003), Vetten in Sarkin (2008), the African Union 52nd session (2012), the UNODC (2017) independent evaluation report in 10 SSA countries, and the review of literature conducted by Reid (2016) where there is a commentary on the status of penal institutions in Africa as a whole, with some SSA countries referred to. With these five, the total of records is 64. Further extensive detail on all records are documented in the Additional file 1: Table S1

### Theme one: the prison physical environment

#### Overcrowding in female prisons

The review highlights variation of degrees of overcrowding and standards of sanitation in prisons located in SSA countries. In a literature review by Reid et al. in 2012 [8] on tuberculosis and HIV Control in SSA prisons, the authors documented outdated physical infrastructure of prisons and severe overcrowding with associated severe health harms. The 52nd ordinary Session of the African Commission on Human and People’s Rights in 2012 also emphasised, given the levels of overcrowding, that SSA prisons were generally not a safe place for pregnant women, babies and young children [32]. In 2013, Matsika et al. [33] reported that in Zimbabwe, up to 15 women were crammed in one tiny cell with their children. Zambian news reporting in 2014 also reported that conditions for pregnant women, mothers and children in prisons were not safe [34]. In 2011, Todrys and Amon [35] conducted in-depth interviews with 46 key informants (government and NGO), 38 adult female prisoners and 21 prison officers in four Zambian prisons (Lusaka Central, Kamfinsa State, Mumbwa, and Choma State), in order to assess perspectives on the health and human rights concerns of female prisoners. Their general conclusion was that: *“women in Zambian prisons live in conditions of severe overcrowding. Zambian prisons are over 300 percent of capacity, and female inmates reported sleeping four to a mattress, packed together in unventilated cells with young children and the sick”* [35]. A later qualitative study by Topp et al. in 2016 in four Zambian prisons (23 female prisoners and 21 prison officers) reported some improvement but with variations in levels of overcrowding in cells across sampled prisons [36]. This was corroborated by findings reported by Malambo in 2016 [37]. In Djibouti, overcrowding was less of an issue for incarcerated women and their children, although conditions remained harsh with poor lighting and heating observed [38].

#### Lack of separate accommodation

The provision of separate accommodation for women with their children where they are housed separately from the main prison population has generally not improved in SSA prisons. In 2017, the United States (U.S) Department of State reported that in Côte d’Ivoire, Madagascar and Senegal, harsh prison and detention centre conditions were described as potentially life threatening due to absence of separate cells for mothers and their children, and with provided accommodation overcrowded, poorly ventilated and without sufficient natural light [22, 39, 40]. The U.S Department of State reported in 2014 that in Benin, Burundi, Côte d’Ivoire, Botswana, Nigeria and Tanzania, while children were permitted to stay with mothers in prison, no separate accommodation was provided for them [23]. In the Ugandan context (Masindi prison) as early as 2001, the AU Special Rapporteur reported on the lack of availability of separate space for mothers with children [41]. In the years 2001, both the Special Rapporteur [42] and Twea in 2013 [43] described Malawian nursing mothers and their children housed in mixed, overcrowded, poorly ventilated and dark holding cells. Little improvement was observed in Malawi or Côte d’Ivoire over time. A study by Baker and the Danish Institute Against Torture (DIGNITY) in 2015 [44] reported that that there was no separate accommodation provided for mothers with children in two large Zambian prisons. Makeshift detention facilities holding many women and their children have been described in Rwanda [45] . Women prisoners in Zambia described concern for their children’s health when sharing accommodation with other prisoners;

*“…I am worried about the children who are here. There was a baby who died. They don’t pay any particular attention to the children. They are mixed in with everyone, they don’t have their own cell or better food…”* [46]*.*

In 2014, the Zambia Times reported that sleeping conditions at Lusaka Central Prison for children were not safe or secure. An officer commanding one of the Central Prisons in Zambia commented;

*“…sleeping conditions at Lusaka Central Prison do not provide incarcerated children with space that is safe and secure… We have people with different kinds of ailments in prisons and children are supposed to be protected at all times… Yet now we can’t find that environment in the prison at the moment…”* [34]*.*

The overcrowded, poorly ventilated and unsanitary conditions in the majority of SSA prisons that mixed the sick and the healthy was reported to exacerbate risk of poor health, and increase risk of infection for mothers and their children [8, 15]. In Cameroon, Kenya, Nigeria and Zimbabwe, prevalence of such ailments as colds, coughs, acute respiratory tract infections, constipation, and rashes among children were attributed to poor environmental health conditions in prisons [21, 33, 47–49]. A Zimbabwean female prisoner described the grave conditions in the remand prison where she was being held before sentencing;

*“… Raising a child in this situation is like living in hell…”* [50]*.*

#### Poor sanitation

There were many reports of the accommodation of women with their children in inhumane, poorly sanitised, ventilated, and in unhygienic conditions across the SSA region [21, 33, 35, 36, 44, 47, 48, 50–57]. More than half of the 27 SSA countries where literature was available, reported poor sanitation in prisons, with little change since 2001. These countries were Chad, Cameroon, Côte d’Ivoire Djibouti, Ethiopia, Kenya, Madagascar, Malawi, Mozambique, Namibia, Senegal, Sierra Leone, Somalia, Uganda, Zimbabwe and Zambia [33, 34, 36, 41–43, 48–53, 58–63]. Reports mentioned insufficient or broken-down toilets or lack of access to toilets especially at night, failure to keep toilets clean through overuse, lack of water or erratic water supplies, and location of the toilet or container in the room accommodating mothers and their infants. In Zambia, a mother shared her experience of such conditions;

“…*You should smell the stench. All the kids are sick, with diarrhoea, and you’ve got this stench coming from the toilet, and someone sleeping with a baby next to it…”* [44].

In Cameroon, Nigeria, Sierra Leone and Zimbabwe, shared buckets in cell corners, often overflowing, were used as toilets, with reports of women prisoners having to use their hands and buckets to dispose of its contents when the drain overflowed and having to remove faeces from the drain [47, 49, 51, 53, 64]. In Zimbabwe in 2003, women prisoners used 25 litre plastic containers especially at night;

*“..By morning the bucket will be a total mess and mothers with babies had to restrain them from crawling on the floor in such a mess… ”* [53].

General hygiene for women and their children across all records was poor. Poor sanitary conditions worsened by the inadequate supply of cleaning detergents and soap were reported in Cameroon, Chad, Côte d’Ivoire, Djibouti, Ethiopia, Ghana, Kenya, Madagascar, Malawi, Mozambique, Namibia, Nigeria, Senegal, Sierra Leone, Somalia, Uganda, Zambia, and Zimbabwe [33, 41–43, 46, 48, 51–53, 55, 56, 59, 60, 62, 64–66]. In 2010, in South Africa, Hesselink and Dastile [67] reported that only one bath, a shower and a toilet were available for all mothers and their children at the Pretoria Correctional Centre. A mother at the Pretoria Correctional Centre commented:

*“…Have to wake up at midnight or early hours in the morning for hot / warm water…”* [67]*.*

One woman shared her experience on the lack of hygienic bathrooms and of safe clean tap water in a Zimbabwean women’s prison:

*“…Toilet sanitisers*^1^ *are scarce. Sinks are not working and there is no running water …”* [63]*.*

In contrast, the United Nations Office on Drugs and Crime (UNODC) in 2014 reported that in South Sudan, basic cleaning products, disinfectants and sanitary napkins were provided by the Juba State prison to female prisoners [68]. Since 2008, NGO and religious organizations were described as providing toiletries to women prisoners in Burundi, Côte d’Ivoire, Sierra Leone, Uganda, Zambia and Zimbabwe [23, 36, 37, 39, 41, 51, 52, 57, 69, 70].

The unhealthy environment exposed children (and their mothers) to gastro-intestinal pathogens. A female prisoner incarcerated in Uganda in 2017 expressed her concern;

*“…The shortage of stable state supply of basic services…such as water makes it more difficult for those who have children in prison … this affects the care given to the children, who have increased risk of diarrheal diseases…”* [62]*.*

In 2017, Makau et al. [48] conducted a cross-sectional study with 202 children and 193 mothers in eight Kenyan prisons. They reported that diarrhoeal diseases and vomiting were common among children in prison. The mothers attributed these illnesses to inappropriate sanitary habits and to the fact that only a small proportion of children had access to treated/boiled drinking water.

#### Mother and baby units

The review highlights where reporting is available, that there is great variation between countries, and even between prisons within a given country. There have been some encouraging improvements, albeit modest and at times temporary. Since 2014, in Ethiopia, Ghana, Kenya and Uganda, a minority of prisons are reported to have separate mother and baby units [41, 61, 62, 71]. South Africa is also a unique case in point. In 2010, a qualitative study using in-depth interviews with a sample of 14 women conducted by Hesselink and Dastile [67] in Pretoria and Johannesburg, described variations in accommodation arrangements for mothers. All mothers and their babies at the Pretoria correctional centre, were accommodated in one communal cell, with only three cots available for infants, while at the Johannesburg female correctional centre women awaiting trial and those already sentenced were housed in single cells (where the mother and the baby share a bed). The Special Rapporteur in 2004 reported on the provision of a mother and baby unit in Durban [72], with the first model Mother and Child Unit attached to the Pollsmoor prison opening in 2011 [73]. The provision of a greater number of mother and baby units in South Africa has been observed, particularly in the Gauteng province. As of December 2014, 16 female correctional facilities out of a total of 22 located in Gauteng have been designed to accommodate both children and their mothers [74].

### Theme two: food availability, adequacy and quality

#### Inadequate food allocation and poor nutrition for children

Nutrition standards in SSA prisons are generally reported to be poor, and thus not only for children imprisoned with their mothers. Generally, this involves the provision of one primarily vegetarian meal per day [18]. Prisons systems generally do not allocate food to children incarcerated with their mothers. Governments in Côte d’Ivoire, Zambia, Uganda and Tanzania were specifically reported to not have an allocation for the care of children [23, 39, 44, 69]. In 2017, Muhangi et al. [62] reported that in Kenyan and Ugandan prison systems, some allocations of financial resources for children were recorded.

Poor quality nutrition and inadequate provision of food for children incarcerated with their mothers was reported in Benin, Cameroon, Chad, Ghana, Kenya, Malawi, Mali, Mozambique, Nigeria, Sierra Leone, Tanzania, Uganda, Zambia and Zimbabwe [33–35, 41–43, 49–51, 57, 58, 71, 75]. In 2011, Todrys and Amon [35] underscored how inadequate nutrition is a serious problem for pregnant women and women with children in Zambian prisons. A Zambian prison officer commented;

“…I get no budget for the children’s food, they must eat their mothers’ food. They are hungry a lot…”[35].

Incarcerated mothers were documented as sharing their allocation of food with their children in prisons located in Cameroon, Chad, Côte d’Ivoire, Ghana, Malawi, Mozambique, Senegal, Sierra Leone, Uganda, Zambia and Zimbabwe [9, 32–34, 41, 42, 44, 46, 47, 49–51, 57, 58, 60, 71]. A Zambian mother commented;

*“…My child is not considered for food—I give my share to the baby (beans and kapenta [sardine]) we eat once a day. The baby has started losing weight and has resorted to breast milk because the maize meal is not appetizing…”* [46].

A prison officer corroborated this statement during an interview;

“…*Yes, we do not provide food for children but the mother shares her portion with the child…”* [37].

Conflicting reports between prison officers and women prisoners were also documented by Malambo in 2016 [37] where Zambian prison officers reported extra provision of rice to breastfeeding mothers and children. Female prisoners in this study denied this. With regard to the provision of adequate protein in the diet, one mother in a Zimbabwean prison commented;

*“…Meat is only part of the diet on important occasions such as the Prisons Day Commemoration...”* [33]*.*

Contrary to the World Health Organization (WHO)/UNICEF guidelines [76] on exclusive breastfeeding for the first six months, in Zimbabwe it was reported that regardless of age, all incarcerated children were required to consume non-breast milk foods as early as possible, to compensate for infrequent and inadequate breastfeeding resulting from their mother’s prison work routine [33]. A 2013 newspaper account in Uganda reported that at Moroto prison, whilst NGOs provided food for children, the majority of incarcerated children were still dependent on their mothers’ milk [70]. A mother expressed the inadequacy of food as follows:

*“…Sometimes our babies go without food. They suckle from morning to evening…”* [70].

For incarcerated mothers unable to breastfeed in Zambia, no baby formula was available [57]. A prison officer in Zimbabwe commented;

*“…The prison tries as much as possible to provide baby food to the children living with their mothers, and some well-wishers have stepped in to supply the food, but it quickly runs out and there is a general shortage. In some cases, the mothers feed on their babies' food because they are also starving…”* [50]*.*

In contrast, and indicating some improvement in nutrition provision and standards, Ethiopian, Kenyan, Namibian and South African prisons were reported to provide additional food for nursing mothers and their children [59, 61, 62, 72]. In Malawi, in 2013 it was reported that on rare occasions soya flour was provided to children [43]. Children at the Luzira prison in Uganda were provided with cow’s milk and vegetables from the prison farm [41, 62]. Variations in provision of special diets for nursing mothers and their children, and the provision of food items such as bananas, fruits and baked goodies like biscuits and banana bread to infants were also noted in South Africa [67]. Most encouraging was that in 2017, Makau et al. [48] reported that out of all 35 female prisons in Kenya, eight prisons provided children with three meals and at least two snacks per day.

### Theme three: provision of basic necessities

#### Inadequate bedding, linen and mosquito nets

Provision of mosquito nets, sheets, cot beds and blankets for infants and children was observed to be inadequate in Cameroon, Ethiopia, Kenya, Somalia, South Africa, Zambia, and Zimbabwe [44, 48, 49, 51, 52, 56, 67]. In Kenya in 2016, incarcerated mothers were reported to be sleeping on dusty and cold floors with their children [48]. In Sierra Leone in 2008, the lack of basic infection prevention and control practices for bedding was observed by AdvocAid [51], which reported that mattresses and blankets were recycled among prisoners, and were filthy and old. A mother incarcerated in Zimbabwe in 2015 described the scarcity of warm blankets for infants;

*“ …You are forced to return to jail within 48 hours after giving birth at public health facilities together with the newly born baby and that is when you get an extra blanket for the baby…”* [52]*.*

#### Inadequate baby clothing, diapers, and baby toiletries

The lack of provision of adequate clean and warm baby clothes, diapers and baby toiletries (for example, baby wipes) was documented in Cameroon, Ethiopia, Kenya, Malawi, Mozambique, Namibia, Sierra Leone, Somalia, South Africa, Tanzania, Zambia and Zimbabwe [23, 36, 42, 44, 48, 51, 56, 58–61, 67, 72]. Reliance on donations by NGOs and faith-based organisations was reported in Burundi, Côte d’Ivoire, Sierra Leone, South Africa, Uganda, Zambia and Zimbabwe [22, 23, 36, 37, 51, 57, 67, 70]. Access was controlled by prison staff, with limited supplies not equitably distributed to mothers per their identified need, and with prison staff taking some supplies for their own families. In Zambia, although NGOs and faith-based organisations provided basins, soap, baby clothes and milk powder, there was no policy or systematic practice to ensure regular or equitable access to such essentials [44]. Similarly in 2008, mothers in Sierra Leone indicated that receiving of these supplies were at the discretion and “good-will” of prison officers [51]. In 2017, mothers incarcerated in Cameroon complained that the supplied clothing for infants was of such poor quality that it was often coarse, unhygienic and unsuitable [49]. Topp et al. in 2016 also described how the lack of clean clothing was a daily struggle for the mothers and their children in Zambian prisons [36]. In 2010, incarcerated mothers at the Pretoria Correctional Centre in South Africa were documented as complaining of inadequate provision of baby clothing given the harshness of winter temperatures, particularly at night [67]. The lack of warm clothing for infants in Kenyan prisons was also documented in 2016 [48].

### Theme four: availability and accessibility of health services for incarcerated children

#### Inadequate prison health care for incarcerated children

Statistics on doctor-to-prisoner ratio or nurse-to-prisoner ratio are not readily available in the SSA region. Within the general population similar statistics pertaining to doctor-to-prisoner ratios are also not easily obtainable. Availability and accessibility to paediatric health care in prison were generally reported to be inadequate in the SSA region, and failing to meet minimum human rights standards regionally and internationally [36, 47, 52, 53, 57, 66]. In Zambia, for example, availability and accessibility of ante-natal care (ANC) was reported as a challenge in 2011 [35], with pregnant prisoners commenting on the lack of medical examination on entry to prison;

“…*I had no initial exam when I came to the facility, even though I am pregnant. There is no special treatment for pregnant women, I take whatever I can…”* [35].

“…*I have not been to the clinic yet, no antenatal care. I went to the clinic once but was told the nurses were not working. Since then I have not asked. I do not feel well, lots of ups and downs…”* [35].

In some countries (for example, South Africa and Kenya), however, encouraging findings were reported [48, 67, 73, 74]. In South Africa, the doctor-to-prisoner ratio was documented as better than in free society. In 2010, Hesselink and Dastile [67] reported on sufficient standards of medical care for incarcerated women and their children in South Africa supported by prison clinics with qualified medical staff. Despite small samples of prisoners interviewed in their study, the majority of incarcerated mothers indicated satisfaction with the quality of prison health care. In South Africa, prison services collaborated with community health care providers in the provision of health care services for the prevention of communicable and non-communicable disease, pregnancy and post-partum care, immunisations, and general health education and promotion for women and their babies. Access to and uptake of immunisation programmes were favourably reported in Zambia, albeit with some restrictions where mothers were not permitted to stay with their infants for health education and promotion after immunization [44]. However, in Malawi in 2013, and in Sierra Leone in 2008, incarcerated children were described as not taking advantage of key under-five services such as immunizations against polio, TB, diphtheria and measles [43, 51].

Based on the data sources available, paediatric health care provision in prison in most SSA countries was reported to be unavailable or lacking key critical resources such as essential medicines, trained medical staff, and specialised care, and overwhelmingly affected by barriers for women to access if available. These countries included Burundi, Cameroon, Chad, Djibouti, Côte d’Ivoire, Ghana, Kenya, Madagascar, Malawi, Mozambique, Senegal, Sierra Leone, Somalia, Uganda, Zambia and Zimbabwe [23, 36, 42–44, 47, 50–52, 54–58, 60, 62, 72]. In Côte d’Ivoire, NGOs sometimes financed prisoners’ medical care [39]. A mother in Zimbabwe said;

*“…Children suffered the most. They did not get good medical care in time. If you asked for help for your child they would tell you hurtful things like ‘Prison has no free medicine…”* [53]

Medicine stock-outs were described by prisoners in Zambia;

*“…my child had a high temperature and cough. She was taken to the clinic by prison officers but there was no medicine for my baby…”* [57].

Access to health care provision in prisons in SSA was further worsened by restricted opening hours for mothers and their children, controlled access by prison guards and negative staff attitudes toward incarcerated children who were acutely or chronically ill in Chad, Cameroon**,** Kenya, Senegal, Zambia and Zimbabwe [22, 36, 44, 47, 53, 54, 65]. In 2015, distressed mothers in Zambian and Cameroon prisons complained of lengthy delays by prison staff to respond to their children’s acute medical needs and how they were denied access to the prison clinic, even in the event of medical emergencies [44, 47]. Delays in medical intervention for ill children and the consequent high risk of child mortality were reported in Zimbabwe by Samakaya-Makarati [53] in 2003. In an interview with IRIN News [50], a prison officer in Zimbabwe said;

*"…I have a feeling that most of the children who die here could have survived if they enjoyed better health facilities… "* [50].

Paediatric deaths caused by delay in access to medical care and general medical neglect were reported in Zambia and Zimbabwe in the years 2003, 2010 and 2015 [44, 53, 57]. A mother in Zimbabwe said;

*“…When you ask, you are sometimes told… ‘This is not home. ‘You knew that you wanted to look after your baby very well. Why did you commit a crime? After two weeks my baby started to show deteriorating health, she couldn’t eat anything. She cried most of the time. I asked to see a doctor, they couldn’t let me see the doctor. So when my family came I asked them to take her…After about a month the baby passed away…”* [53]*.*

#### HIV prevention, treatment and care for incarcerated children

In June 2016, the United Nations General Assembly agreed that ending AIDS by 2030 requires a fast–track response (*Political Declaration on HIV and AIDS: On the Fast Track to Accelerating the Fight against HIV and to Ending the AIDS epidemic by 2030*) [77]. Despite this, HIV testing, TB screening and treatment coverage in SSA prisons were generally reported to be weak, with limited or no provision of services for prevention of mother-to-child transmission (PMTCT) of HIV in prisons in Angola, Ethiopia, Lesotho, Malawi, Mozambique, Namibia, Swaziland, Tanzania (including Zanzibar), Zambia and Zimbabwe [18, 32, 35]. This lack of sufficient PMTCT facilities was documented as contributing to increased rates of mother-to-child transmission of HIV within prisons located in SSA countries [32]. This is concerning given that these countries are among the most HIV-affected countries globally. In Zimbabwe, the Network for People Living with AIDS (ZNNP+) in 2010 described how children already diagnosed with HIV would accompany their mothers into prison [50]. The report stated;

***“…****Many HIV positive children are dying in prison because they are failing to access treatment, and it is the responsibility of the government to make anti-retroviral therapy accessible to them…”* [50]***.***

South Africa represents a positive example where PMTCT services are scaled up and available in prisons. Social work services and psychological services were also documented to be available upon request or referrals after proper assessments [74].

## Discussion

This scoping review represents a first step toward mapping available literature on the health situation and unique rights violations of children incarcerated with their mothers in SSA prisons. There is a paucity of published evidence on this vulnerable population. The information found in this scoping review underscores the grave circumstances for infants and young children incarcerated with their mothers. Incarcerated children are a hidden population in SSA prisons who continue to be ignored in terms of prison resource allocation for basic needs such as safe and clean sleeping and living areas, basic nutrition, ventilation and light, adequate clothing, sanitary products, and pediatric medical care. The review highlights that similar to adult prisoners in SSA, they are incarcerated in situations which do not comply with international mandates in treaties ratified in nearly all SSA countries.

Prison conditions in the SSA region are harsh for all prisoners. Children like the general prison population are adversely affected by lack of separate accommodation and individual sleeping space, overcrowded cells, inadequate bedding, hygiene and sanitation, and lack of clean and warm clothing, food and safe drinking water. Harsh prison environmental conditions serve to exacerbate the spread of common respiratory and gastro-intestinal conditions, as well as diseases such as TB and malaria. This review, which focused on the past 18 years, underscores little improvement over time, with exception of South Africa.

Prison health for mothers and incarcerated children is generally dismissed or allocated a low priority by SSA government policy makers and prison health programmers perhaps due to their low numbers in comparison to the large male prison population [18]. International decrees as previously mentioned mandate equivalence of care in prison, to that provided in the community, and access to equitable health services for people in prisons free of charge (the Mandela Rules, Rule 24.1 [10]. Most encouraging however, is that South Africa as key forerunner in the region has significantly improved its prison conditions for women and their children, alongside upscaling maternal and child health (MCH) care services in prisons [74]. Of note in other SSA countries, is the lack of recording of incarcerated babies and children, including the rates of pregnancies in prisons, and poor provision of key SRH services for women and their children. There is a reported lack of pediatric health care services in prisons, and if services are available, barriers to access exist and result in low uptake. Medical care provisions (with exception of those in South Africa) for women and their children were documented as poor, and characterized by lack of essential medicines, frequent medicine stockouts, negative staff attitudes toward the incarcerated children and their medical needs, prison officers who are not health professionals controlling access to medical care, and restricted uptake of incarcerated children to immunization programmes. The reported poor provision of pediatric medical services and lack of access, often dictated by prison officials, not medically trained, contributes to very poor child health and risk of child mortality whilst incarcerated with their mothers in SSA. Infant deaths were reported in some countries (for example Zambia, Zimbabwe), as consequence of medical neglect and denial of medical care.

Prisons in the SSA with exception of South Africa are however generally failing to address PMTCT of HIV in prisons [18]. This is despite the fact that AIDS and TB are among the main causes of death in prisons, with prisoners five times more likely to be living with HIV than adults in the general population [19]. Incarcerated women are at higher risk of acquiring HIV, TB and other infections in prisons, than men, and also have a higher prevalence of HIV, and an even higher prevalence than women living in the community [78]. This may result in a higher proportion of children born in prisons being at risk of HIV infection compared to children born in the community. The limited access for women (and their children) to ANC, labour and delivery services and anti-retroviral treatment (ART) whilst incarcerated in SSA poses a serious challenge to PMTCT of HIV. The inadequacy of PMTCT services in prisons contributes to infants being at high risk of contracting HIV during pregnancy, delivery or breastfeeding. Restricted access of infants to their mothers for feeding in some prisons (for example Zimbabwe) and the lack of adherence to good infant feeding practices heightens risk of transmission [33]. In resource-poor settings, when formula feeding is not a viable option, women living with HIV are advised to exclusively breastfeed (rather than mixed feeding) in the first six months [79, 80]. This was not the case in certain countries such as Zimbabwe where mothers are required to work in prisons during the day, thus interrupting their infants’ access to breastmilk. In May 2017, the UN Commission on Crime Prevention and Criminal Justice (CCPCJ), adopted a resolution [81] requesting Member States in close cooperation with UNODC and other relevant United Nations entities and other relevant stakeholders, to increase their capacity to eliminate mother-to-child transmission of HIV, and support HIV prevention and treatment programming in prisons, particularly in countries with a high-burden TB/HIV coinfection in the SSA region.

We recognise the limitations of this review centring on the relative lack of data sources with only 27 countries represented. Strengths centre on the thoroughness of the review approach in terms of its multi-layered strategies to locate all forms of information. The wide timespan of the mapping exercise (18 years) with sporadic documentation of prison conditions makes it difficult to establish whether the situation has improved or deteriorated. The gathering of strategic information through surveillance, country situational assessments and routine monitoring and evaluation, and investment in academic research in SSA prisons at country level warrants improvement.

## Conclusions and recommendations

This review highlights the grave situation of infants and children incarcerated with their mothers in SSA prisons. While all prisoners in the region suffer from poor prison conditions, children are particularly vulnerable to the health impact of these conditions. The reported paediatric morbidity and mortality associated with such sub-standard prison conditions is deeply concerning and in contravention of all international mandates for the rights of the child and the right to health and standards of care. Imprisonment of women, particularly pregnant women and women with children, should always be a last resort, and suitable non-custodial alternatives should be made available whenever possible (Bangkok Rules) [13]. The review further highlights the need for enhanced monitoring and evaluation of children’s situation in prisons, along with increased donor and governmental resources allocation for services to meet basic needs of incarcerated children and paediatric health care. In particular, the documentation of children in prisons should be mandated for all countries so that their presence is recorded and therefore the conditions of their incarceration could be reviewed.

## Additional file

Additional file 1: Summary of Records. The scoping review charting of records. (DOCX 93 kb)

## Abbreviations

ACRWC: African Charter on the Rights and Welfare of the Child
ART: Anti-retroviral treatment
AU: African Union
CCPCJ: Commission on Crime Prevention and Criminal Justice
DIGNITY: Danish Institute Against Torture
HIV: Human immunodeficiency virus
MCH: Maternal and child health
NGO: Non-governmental organizations
PMTCT: Prevention of mother-to-child transmission
SADC: Southern African Development Community
SSA: Sub-Saharan African
TB: Tuberculosis
UN: United Nations
UNICEF: United Nations Children’s Fund
US: United States
WHO: World Health Organization

## Footnotes

1 Toilet sanitisers are disinfectants or detergents used to disinfect toilets against such infectious bacteria as *E. coli*, Shigella, Streptococcus, and Staphylococcus.
