# Supplementary material for: ‘Mankind owes to the child the best that it has to give’: prison conditions and the health situation and rights of children incarcerated with their mothers in sub-Saharan African prisons
Source: BMC Int Health Hum Rights. 2019 Mar 5;19:13. doi: 10.1186/s12914-019-0194-6 (PMC6402132; doi:10.1186/s12914-019-0194-6)
Supplement: Supplementary file 1 — Summary of Records. The scoping review charting of records. (DOCX 93 kb) [file 12914_2019_194_MOESM1_ESM.docx]

**Supplemental Table ‘Summary of Records’**

| **Authors** | **Title, Journal, Year of Publication, Volume, Pages** | **Aim** | **Location** | **Method of Study** | **Results** | **Conclusion** |  |
| --- | --- | --- | --- | --- | --- | --- | --- |
| **Journals** | | | | | | | |
| Topp SM, Moonga CN, Mudenda C, Luo N, Kaingu M, Chileshe C, et al. | Health and healthcare access among Zambia’s female prisoners: a health systems analysis  Int J Equity Health. 2016; 15:157. | To identify and examine the interaction between structural, organisational and relational factors influencing Zambian women prisoners’ health and healthcare access. | Zambia | Qualitative Research  In-depth interviews of 23 female prisoners across four prisons, as well as 21 prison officers and health care workers. | A high proportion of women reportedly struggling to look after young children in prison.  Seven of the 23 inmate respondents (30 %) had a child living with them at the time of study.  Children’s ages ranged from 3 months to 4 years old.  Inmates consistently described their children’s acute vulnerabilities  Finding sufficient and appropriate food and clean clothes for infants was described as a daily struggle.  All women described inappropriate types and quality of food for infants and described the difficulty of maintaining breastfeeding when their own nutritional status was so weak; finding supplemental milk when they were unable to breastfeed appeared to be a matter of chance.  Several women described having (unsuccessfully) requested transfers to prisons nearer their family in order to access their support  Two women reported the ‘buffering’ effect of belonging to mixed group since group members would sometimes share food.  Some described the impossibility of asking other women for food without something to exchange.  Church and NGO donations were fundamental to fulfilling their children’s basic needs, although even these were often insufficient.  All reported challenges in accessing timely health services for their children.  Overcrowding with 8-10 women sharing a single cell meant for inmates per cell.  Insufficient toilets, broken toilets or lack of access to toilets (at night) with inability to keep toilets clean, through overuse and lack of cleaning products mentioned for all sites.  Scarcity of laundry soap was also described.  . | Prisons are ill equipped to accommodate and provide for the basic needs of children. Inadequate nutrition results in stunted growth and exposes children to common ailments.  Civil rights organizations should advocate for the cause of children so that at least provision is made for children within governments budget. |  |
| Reid SE, Topp SM, Turnbull ER, Hatwiinda S, Harris JB, Maggard KR, et al | Tuberculosis and HIV control in sub-Saharan African prisons: “thinking outside the prison cell.” J Infect Dis. 2012;205: S265–S273. | To describe the challenges inherent in current approaches to tuberculosis control in prisons and consider the alternatives. | Africa | Literature review | Female inmates face the same overcrowding and unsanitary conditions as males that contribute to the spread of infectious disease and poor health.  Female inmates experience unique health-related challenges, such as menstruation, pregnancy and childbirth, care of children inside and outside prison, and extreme violence and (often sexual) abuse by prison officers and male prisoners | Unconducive prison environment exacerbates the spread of communicable diseases |  |
| Todrys KW, Amon JJ. | Health and human rights of women imprisoned in Zambia. BMC Int Health Hum Rights. 2011; 11:8. | The main objective was to document and respond to specific human rights issues, monitor human rights conditions, and assess human rights protections in Zambian prison. | Zambia | A mixed-methods study including in-depth interviews with 38 adult female prisoners and 21 prison officers in four Zambian prisons Key informant interviews with 46 officials from government and non-governmental organizations and a legal and policy review. | Women prisoners live in conditions of severe overcrowding. Prisons are over 300 % of capacity, inmates sleeping 4 to a mattress, packed together in unventilated cells with young children and the sick  Both prisoners and prison officials reported insufficient and nutritionally inadequate food. Prisoners rely on their relatives for supplementation or trade work for food.  No provision of basic necessities as soap, toothpaste, or sanitary pads.  Of Zambia's 86 prisons, only 15 had any health clinic or sick bay.  For those at prisons without a clinic–and for those with more serious medical conditions at those with a clinic–access to care is controlled by medically unqualified and untrained prison officers.  Both prisoners and prison officers, indicated a lack of adequate prison staff for the transfer of sick prisoners, inadequate vehicles for transportation and fuel, and security fears prevent inmates from accessing medical care outside of prisons, in some cases for weeks after they fall ill.  Inmates reported delays of up to a month waiting to go to the clinic.  Incarcerated pregnant women described inadequate, and in some cases non-existent care.  For some ANC care existed but did not meet international standards.  No PMTCT program under the prison medical directorate, though PMTCT programs have been scaled up in recent years for the general population.  Inadequate nutrition is a serious problem for pregnant women and women with children in prison.  The Prisons Service allocates no food to children who live with their mothers in prison facilities. In situations where women are unable to breastfeed, the prison does not offer infant formula.  HIV testing and treatment was offered at six prisons nationwide with the assistance of an NGO, and as of March 2011, prison-based TB screening and treatment offered only at three prisons nationwide as part of a pilot programme.  HIV testing not mandatory prenatally and Zambia Prisons Service policy prohibits compulsory HIV testing.  Mandatory testing was conducted prenatally for all pregnant women.  Female inmates previously held in police custody, reported physical and sexual abuse | Access to health care by female prisoners was a challenge due to a number of factors identified in the study. The issue of inadequate nutrition and lack of a budget to cater for children affects both growth and developmental milestones of the children |  |
| Todrys KW, Amon JJ, Malembeka G, Clayton M | Imprisoned and imperiled: access to HIV and TB prevention and treatment, and denial of human rights, in Zambian prisons. J Int AIDS Soc. 2011; 14:8. | To better understand the relationship between prison conditions, the criminal justice system, and HIV and TB in Zambian prisons | Zambia | Mixed method  facility assessments and in-depth  interviews with 246 prisoners and 30 prison officers at 6 Zambian prisons; a review of Zambian legislation and  policy governing prisons and the criminal justice system; and 46 key informant interviews with government and  non-governmental organization officials and representatives of international agencies and do | Some health facilities had very little medical capacity beyond distributing paracetamol  According to staff and prison officers, in prisons without a medical clinic – and for prisoners with more serious medical conditions requiring advanced care - access to care is frequently controlled by medically unqualified and untrained prison officers who evaluate and determine if medical visits to community health facilities are necessary.  Prisoners and prison officials blamed the lack of sufficient prison staff, transportation and fuel.  Security fears, lengthy delays in the transfer of sick prisoners to medical care outside, in some cases for days or weeks after they fall ill.  Inmates consistently reported that the requirement to work long hours in farms frequently prevented them from accessing necessary medical care.  Variability in Voluntary Counselling and Testing (VCT) uptake with less women likely to undertake testing. | Barriers to access to medical care was worsened by lack of medically trained prison staff and administrative bottlenecks such as security fears, lack of transport and fuel. |  |
| [Danish Institute Against Torture](https://stoptorture.today/publications/) (DIGNITY) | Conditions for women in Detention, in Zambia Needs, vulnerabilities and good practices  Dignity Publication; Series on Torture and Organised Violence No. 12 -Copenhagen, 2015.<https://www.jobakeronline.com/articles/conditions-for-women-in-detention-in-zambia-needs-vulnerabilities-and-good-practices/> Accessed 9 April 2018 | To identify the major needs and risks that are common to imprisoned women in Zambia, and the way that this impacts them. It also aimed to find challenges and promising practices that are common in the management of female detainees, with international standards as a benchmark. | Zambia | Mixed methods approach was used.  Observational visits were conducted to two of the larger prisons operated by Zambia Prisons Service (ZPS) and one maximum security men’s prison.  Semi-structured in-depth interviews were conducted with detainees, as well as meetings and structured interviews with prison staff and others working in and around prison communities in the country. 39 persons in total. | Women with young children in prison complained of poor conditions making it impossible for them to keep their children clean, healthy and well fed.  Staff most often raised health and hygiene concerns during interviews, particularly the lack of ventilation and the unsanitary toilets, washing and cooking facilities, and expressed particular concern for pregnant women, new mothers, and ‘circumstantial’ (accompanying) children.  Zambian prisons remain designed for male inmates, with no special provisions suitable for women detainees, such as separate accommodation for mothers and children.  Not all have sheets and few have mosquito nets and in 2009, two pairs of mothers and babies were reported sharing a single mattress in Lusaka Central.  There is no living space or play space for children once the mattresses are laid out.  Mothers with infants share the same facilities with other inmates, with no special provisions for nursing inmates and their infants, nor childcare provisions.  One prison had an only flushing toilet located in the room occupied by mothers and infants, and at least one mattress was located less than two meters from the toilet, which sometimes gets blocked.  The Prison Service does not regularly provide inmates with basic necessities, including soap.  Food was particularly insufficient for pregnant women and those with children.  Food rations often allocated to inmates in groups, with no extra rations for children and had to be shared.  Clothing, soap and detergent were not provided for these children, and most provisions donated from church organisations and NGOs.  HIV treatment, and care for accompanying children was reported as a direct barrier to care, because prison officers without medical training would sometimes deny this escort if they did not consider an inmate’s condition (or the condition of her accompanying child) to be serious enough.  Offender Management staff try to source essential items for new mothers from outside organizations, including clothes and diapers for babies but does not meet basic needs of all women.  Regular mother-and-child clinic visits take place in larger prisons for vaccinations and check-ups for children aged below four-years-old, commendably in line with WHO recommendations, as in the broader community.  Inmate mothers not permitted to join the routine group information session that is given afterwards, should they wish to  Care is otherwise very limited for young children who are detained with their mothers, and mothers commonly reported the distress of having so little control over their children’s poor health, while feeling the full burden of it.  Tend to access check-ups only if there are clear signs of a problem.  Conditions are grossly inadequate, place the lives of the women and their children at risk, and breach a range of international obligations, particularly the right to health.  Staff report that a large percentage of the children who live with their mothers in prison are over two-years-old, and there are no state provided facilities or provisions for children.  Mothers are worried about their children’s health and hygiene, and their safety among inmates and (often impatient, unsympathetic) | Adequate nutrition in terms of both quality and quantity are critical for the development of a child and building the immune system. Holding of children in overcrowded, unhygienic and unventilated cells predisposes them to infectious conditions such as Tuberculosis. Access to paediatric ARVs should be addressed as a matter of urgency. Medically trained staff should be hired to improve the quality of care given to children |  |
| Sarpong AA, Otupiri E, Yeboah-Awudzi K, Osei-Yeboah J, Berchie GO, Ephraim RKD | An Assessment of Female Prisoners’ Perception of the Accessibility of Quality Healthcare: A Survey in the Kumasi Central Prisons, Ghana. Ann Med Health Sci Res. 2015; 5:179–184. | To explore the background characteristics of female prisoners and how it influences their assessment of the quality of accessible healthcare in the Kumasi Female Prison. | Ghana | Descriptive, cross-sectional study with 39 female prisoners. Interviews and questionnaire administration of 12point scale inventory. | Skin infections and bed bugs were reported in the overcrowded cells.  Health service provider in prison had not attended any refresher course or training  Inmates with babies are retained and stayed until first birthday of the child after which social welfare or the family take custody of the child.  Prison health caregiver was trained as a village health worker to provide health care service in places where there are no doctors. | Although children are retained with their mothers in prison until the 1^st^ birthday, the prison environment has inadequate nutrition availability, and accessibility to health care remain major concerns. |  |
| Solomon OJ, Nwankwoala R | The plight of female prisoners in Nigeria and the dilemma of health rights violations. Asian J Soc Sci Humanit 2014; 3 152-61 | To discuss some of the main challenges faced by female inmates in terms of health and other social issues, and to suggest possible solutions. | Nigeria | Literature review | Irrespective of gender, correctional facilities described as having inhumane conditions, with over-crowded leading to deplorable health situations  Problems with provision of quality health care include inadequate health personnel, facilities and systems, lack of health care policies and standard operating procedures.  An increase in the number of women inmates giving birth in prisons and yet not all prisons are equipped or prepared to handle growing baby population.  It is not uncommon for women in prison to discover that they are both pregnant and HIV positive.  Most toiletries are provided by NGO and religious organizations  ANC rarely given | The inadequacy of and in accessibility of health facilities for female inmates and their children compromises their health. The situation is further compounded by inability of prisons to handle growing baby population. |  |
| Nangia EN, Fontebo HN. | Treatment of Female Offenders in Prison: The Case of Cameroon.  Int J Humanit Soc Stud. 2017; 5:318–24. | To document and examine the treatment of deplorable prison conditions from female prisoners’ perspectives | Cameroon | Qualitative  Focus group discussion with 10 female inmates and employed purposive sampling | Poor diet  Toilets either bad and unfit for use or very unclean which caused inmates to use buckets.  No supply of water which exacerbate the situation.  Overcrowded due to limited space and obstructed ventilation.  No toiletries like rolls, sanitary towels and pads.  Women offenders with babies in prison, it is their own responsibility to take care of their babies.  No special facilities exist to support babies.  Babies sleep on same beds or mats and eat same food like their mothers. | The non-availability of basic supplies such as soap, clothing and adequate bedding exposed children to childhood illnesses such as diarrheal diseases and common colds. |  |
| Linonga-Fontebo H N, Rabe M | Mothers in Cameroonian Prisons: Pregnancy, Childbearing and Caring for Young Children. Afri Stud. 2015; 74: 290-309. | To assess effectiveness of policies in addressing the needs of female prisoners in Cameroon in relation to pregnancy, childbearing and rearing; and to identify strategies that  female prisoners in Cameroon employ in trying to cope with pregnancy, childbearing and rearing | Cameroon | Qualitative  Interviews with 18 female inmates, 13 of whom are mothers (and therefore only their experiences are reported in this article), ﬁve prison staff members and two representatives from a non-governmental organisation (NGO) involved in prisons | Female inmates interviewed alluded to poor health conditions in the detention of small children, leading to ailments such as colds, coughs, constipation, rashes and difﬁculties with breathing.  They called for food, drugs, warm clothing and toys to be made available.  Prison authorities control the environment of the mother and the child without taking responsibility for the well-being of the child.  There were occasions when the baby’s health is at risk: | Regional, continental and international bodies should monitor implementation of policies that pertain to children so that governments are held accountable for inhumane conditions. |  |
| Muhangi, A., Munene, A., Ssibetya. B. N. | Innocent prisoners: Early childhood care and development of young children living with their mothers in prison in Uganda and Kenya.  [Childhood in Africa Ohio University](https://www.ohio.edu/global/cis/african/upload/Childhoold-in-Africa-V4-I1-2017-Spring.pdf) 2017; 4, 11-22 | To examine the care context for young children living with their mothers in Luzira Prison in Kampala Uganda and Langata Women’s Prison in Nairobi Kenya. | Kenya and Uganda | Qualitative data was collected through focus group discussions in depth interviews and observations. In depth interviews with 17 pregnant mothers and mothers with children in prison as well as the 6 prison officers, 2 children's officers and 3 staff from NEST Kenya and Family of Africa in Uganda were conducted. | Langata Women Prison has a welfare department, which caters for both the welfare needs of the mother and the child, health care needs, and nutrition.  Experiences of mothers living with their children revealed mixed feelings.  Langata prison is the only prison in Kenya with a day care center. In other prisons the children spent the day in the wards.  The prison owns a farm that provides food like vegetables and cows that provide milk for the babies while Uganda prison services caters for the nutrition and health needs of all inmates including the children.  Insufficient efforts to ensure child protection in the prison.  Limited establishment of preventive measures like specialized medical care and structures for children. | Availability and accessibility of preventative services and specialized medical care structures should be complimented by public health facilities outside of prison. |  |
| Makau M. N, Ochola S, Mbithe D | Feeding Practices of Children Aged 0-59 Months Accompanying Incarcerated Mothers in Selected Women’s Prisons in Kenya. Open Nutr J. 2016; 11:1–10 | To establish feeding practices of children 0-59 months incarcerated with their mothers in selected women’s prisons in Kenya. | Kenya | A cross-sectional analytical study conducted on an exhaustive sample of 202 children and 193 mothers, drawn from a sample of eight out of the 35 women prisons in Kenya using structured researcher-administered questionnaire for mothers and children. | 69.4% of infants less than 6 months; were exclusively breastfed.  At 1 year was 88.5%.  Continued breastfeeding for two years or beyond among children 18-23 months old was slightly more than half (52.2%) of the children.  Most of the children between 6-8 months old, (92.3%) had been introduced to solids, semi-solids or soft foods.  87.5% of the underweight children were those not consuming the recommended 4 or more food groups.  Children not meeting the recommended minimum acceptable diet in were more likely to be underweight.  91.7% of the children who were sick were also underweight.  In all the prisons visited the children were served with 3 meals and at least 2 snacks per day  Most common childhood illnesses reported by the majority of mothers were those related to the acute respiratory tract infections (ARI).  Child ill conditions were reported to be associated with harsh prison conditions such as overcrowding and exposure to extreme temperatures especially at night.  Langata women prison was the only facility with beds in the cells  Children lacked warm clothing exposing them to excess cold weather at night and during cold seasons, dusty and cold floors within the cells; congestion in some facilities  Diarrhoeal diseases and vomiting were also common among the children and this was attributed to lack of hand hygiene practice with the mothers.  Only a small proportion of children had access to treated/boiled drinking water and caretakers reported the failure to wash hands before feeding the children.  The use of soap was found to be uncommon in the practice of washing of hands.  In the event of sickness, 91.7% of the children were taken to the prison dispensaries for medical care and each prison had own dispensary situated on the male (main) wing of the prison except in the case of Langata prison which is purely a women’s prison.  Majority of mothers were satisfied with the quality of health care within the prison dispensaries and the civilian referral hospitals. Health care availability, accessibility and quality. | The prison routine and tasks mothers have to perform take mothers away from their children making exclusive breast feeding as recommended by WHO difficult to maintain Children were being given a sufficient diet in terms of quantity and quality and this is a good practice that should be adopted and replicated in other SSA prison settings |  |
| Vetten L. | The imprisonment of women in Africa. In: Sarkin J, editor. The imprisonment of women in Africa. Cape Town: HSRC. Wall Street; 2008. p. 134–54. | To assess human rights in African Prisons. | Several African Countries within SSA that included Benin, Ethiopia, South Africa, Zimbabwe Mozambique, Uganda | Literature review | Children are imprisoned with their mothers in Benin, Ethiopia, South Africa, Zimbabwe and Mozambique.  In South Africa children may remain with their mothers until the 5^th^ birthday.  In Ethiopia children were allowed to remain in prison up to the age of 18 months but an African Union Commission visit to the country in 2004 found children in prison as old as eight years and they were not going to school in one prison  In Uganda some babies were reported to be getting the same food as that of their mothers | The presence of circumstantial children in SSA prisons underscores that governments cannot continue to turn a blind eye in the provision of basic needs in their budgetary allocations. |  |
| Matsika, A.B., Muridzo, N.G., Nyanguru,A.C., & Dzingirai, V. | Innocent inmates: The case of children living with incarcerated mothers in Zimbabwe’s Chikurubi Prison.  Journal of Social Development in Africa, 2013;  28(2), 73–92. | The objective was to determine the extent to which the varying needs of the children in joint incarceration with their mothers were being addressed | Zimbabwe | Qualitative utilizing in-depth interviews with 15 mothers accompanied by their children and 4 key informants (KI) namely the Chikurubi Female Prison officer in-charge, the Prison Clinic matron and two rehabilitation officers - one from the Zimbabwe Prison Services (ZPS) at Chikurubi maximum female prison | Physical settings of Chikurubi Female Prison where incarcerated nursing mothers stayed with their young children were very restricted in terms of space as all the 15 mothers were squashed into a single cell together with their children  Cells restricted the babies’ achievement of critical milestones such as crawling and walking.  Mothers indicated that some of the children were constantly coughing and sneezing due to overcrowding, the cold weather and the use of very dirty and dusty blankets  Prison section that housed the nursing mothers and their babies received erratic supplies of clean and safe tap water and mothers resorted to fetching water for consumption from an unprotected shallow well in the prison garden.  Children exposed to an unhygienic living and playing environment that had burst and overflowing sewer pipes.  Mothers acknowledged that the crèche at Chikurubi Female Prison served to fill a critical gap in regard to their children’s access to early education and play time.  Incarcerated nursing mothers had no control over the type, quantity, quality and frequency of provision of food.  Exclusive breast feeding until 6 months old is recommended.  At Chikurubi Female Prison, all the children, regardless of age, were obliged to commence on supplementary feeding at the earliest possible convenience, to compensate for the infrequent and inadequate breastfeeding.  There are no special or separate food preparation facilities for babies and thus the children received most of their rations from the common pot.  Children are fed on mothers’/adult diet that was unbalanced and lacked diversity  Diet consisted of porridge for breakfast, sadza and a relish of leaf vegetables for lunch, and again sadza and leaf vegetables or beans for supper with no gravy (which would normally go with sadza)  Porridge was rarely served with sugar, and this made it very difficult to feed the babies such meals  On rare occasions prison authorities provided peanut butter and meat in their diet. The food provided to the babies was observed to be clearly inadequate and inappropriate for their needs.  At the crèche the children were provided with a more nutritious and diversified diet comprising of porridge with peanut butter, rice, beans, sadza, kapenta, potatoes, mince and donated bread once a week  Feeding timetable followed the normal prison routine | Unhygienic physical environment at the female prison predisposed children to various ailments. WHO policy advocates exclusive breast feeding of infants up to the age of six months unless if contraindicated. Mixed feeding before the age of six months is at variance with international and regional policy. Babies born in prison or accompanying their mothers should not be deprived of exclusive breast feeding more so where there is inadequate and poor nutrition. |  |
| [Hesselink](https://scholar.google.com/citations?user=MmzzjKUAAAAJ&hl=en&oi=sra),A., & Dastile, NP | The reality of babies and toddlers behind bars.  Acta Criminologica: Southern African Journal of Criminology, 2010 (Special Edition 1), 65–79. | The aim of the research article was to explore and describe mothers’ and babies’ / toddlers’ experience of imprisonment within the South African correctional environment. | South Africa Pretoria and Johannesburg female correctional centres | Qualitative interviews with ten females (five unsentenced and five sentenced) from the Pretoria Correctional Management Area and four (two unsentenced and one sentenced) respondents from the Johannesburg female correctional centre conducted. | Mothers interviewed in Pretoria and Johannesburg correctional facilities indicated that adequate medical services were available and facilities were adequately staffed with qualified medical provided for both themselves and their children.  Inadequate cots described in Pretoria, with mothers sharing with children.  Variations in conditions between the two prisons. At the Pretoria correctional centre, the babies / toddlers only receive bananas, while at Johannesburg the babies / toddlers receive a variety of fruits and on Fridays “baked goodies” like banana bread and biscuits  Cold temperatures at night.  Clothing, bottles, pacifiers, nappies, toiletries (i.e. powder and creams) and toys are mostly donated by organisations; non-governmental organisations and individuals, and all the mothers stated that there is a severe shortage of baby baths; walking rings; nappies; blankets; milk formula; toiletries and clothes (new born and other clothes. | In the case of SA, medical facilities and qualified health providers provided care for the inmate mothers and their children. The diet was also balanced. Insufficient basic necessities were reported by the mothers despite supplies by philanthropic organizations and individuals extending a helping hand. |  |
| Samakaya-Makarati. J. | Female prisoners in “male “prisons. In: Musengezi C, Staunton I, editors. A tragedy of lives women in prison in Zimbabwe. Harare: Weaver Press; 2003. p. 11–22 | To explore perceptions and experiences of women inmates accompanied by their children in prison | Zimbabwe | Qualitative  Document review  Interviews with female prisoners (n=14) | Women complained of poor quality and quantity of food they got in prison.  There was no separate diet for children and infants had to share their small portion of food with their accompanying children.  Inadequate health care and medicines for ill children  Negative staff attitudes that resulted in delayed care for the children | Staff attitudes were a direct barrier to access to health care resulting in delays for the children in accessing medical care.  In adequate health care and medicines compromised the health of the children. Civil rights organizations should play a key role in monitoring prison practices to ensure the voice of the defenceless children and their rights are upheld. |  |
| **Evaluation Report** | | | | | | | |
|  | United Nations Office on Drugs and Crime (UNODC). Final Independent Evaluation of Project XSS V02 HIV Prevention, Treatment, Care and Support in Prison Settings in Sub Saharan Africa. UNODC Vienna; 2017. <https://www.unodc.org/documents/evaluation/Independent_Project_Evaluations/2017/XSSV02_independent_project_evaluation_report_2017.pdf>  Accessed 24 May 2018. | The goal was to conduct a Final Independent Evaluation of implementation of project XSS V02 fully in line with set UNODC Norms, Standards, Templates and Guidelines. | Angola, Ethiopia, Lesotho, Malawi, Mozambique, Namibia, Swaziland, Tanzania (+ Zanzibar), Zambia & Zimbabwe) | Qualitative document review, interviews with stakeholders (n=49) Core Learning partners prison wardens (n=64)  16 Focus group discussions with stakeholders (n=64) on line survey with prison wardens (n=6) | Children accompanying their mothers are neglected in HIV Prevention Treatment Care and Support (PTC&S) programming prisons. | A comprehensive package of health care for children accompanying their mothers in prison needs to be urgently addressed to ensure continuity of care and prevent emergence of drug resistance strains of HIV in children |  |
|  | United Nations Office on Drugs and Crime (UNODC). SDNX06. Assisting the Process of Prison Reform in South Sudan. UNODC, Vienna, 2014. https://www.unodc.org/middleeastandnorthafrica/en/project-profiles/sdnx06.html. Accessed 14 May 2018. | The objective of the project is to assist the process of prison reform in South Sudan by building leadership capacity within the prison service to strengthen their ability to manage the prison population more effectively and more humanely | South Sudan | Desk review of different types of documents prepared by the of reports and other sources semi-structured interviews with National Prisons Service South Sudan (NPSSS), United Nations Office of Drugs and Crime (UNODC), United Nations Mission in South Sudan (UNMISS), Bureau for International Narcotics and Law Enforcement Affairs, Government of the United States of America (INL), the UN Office for Project Services (UNOPS) | The independent evaluation report noted the following  Specific attention had been given to improve the health and hygiene situation of female prisoners, their children and other more vulnerable groups through supplementary feeding in ten states (26 prisons),  Female sections of the ten state prisons were supplied with blankets, basic cleaning products and disinfectants to improve hygienic conditions, and sanitary napkins were given to female prisoners in Juba state prison | Overall, the challenges in the country impacts even on correctional facilities ability to provide for both mothers and their children |  |
| **Media Reports** | | | | | | | |
| Agomoh U. | Assessment and Treatment of Female Prisoners in Africa. United Nations Asia and Far East Institute for the Prevention of Crime and the Treatment of Offender (UNAFEI), 2003. related:www.unafei.or.jp/english/pdf/RS_No94/No94_VE_Agomoh1.pdf. Accessed 24 Apr 2018. | To critically analyse how the African continent in which prisons rank low on various lists of priorities has failed to adequately address the plight of incarcerated women. | Several African countries including Nigeria but no list of the countries provided. | Qualitative and quantitative. No numbers provided. | 9.3 % of the female prisoners interviewed were pregnant while 7.8 % delivered their babies while in prison.  Lack of adequate facilities for pregnant female prisoners and those with their babies in prison including pre-natal, post-natal and creche facilities, etc.  78.9 % reported that the babies with their mothers in prison enjoy medical attention in prison but 21.1% reported that this was not the case.  No separate feeding arrangement is made for pregnant women and nursing mothers.  Pregnant and nursing mothers in prison feed on the same menu and budget as every other prisoner | The dearth of information and lack of visibility of the needs of children accompanying their mothers in prisons results in their needs being overlooked.  This is despite regional and international protocols that most governments have ratified within SSA governments. |  |
|  | Newsday. Chikurubi Female Prison sitting on health bomb [Internet]. NewsDay Zimb. 2017 Available from: https://www.newsday.co.zw/2017/03/chikurubi-female-prison-sitting-health-bomb/  Accessed 26 May 2018 | To conduct a tour of the prison complex and explore perceptions of female inmates on their living conditions | Zimbabwe | Qualitative  Interview with prison physician and female inmates at Chikurubi maximum women prison during a tour of the facility by Parliamentary Portfolio Committee on Women Affairs Numbers not provided | Apart from the health challenges, inmates complained about the poor food that was being served to them and appealed to donors to help in improving their diet.  No cot beds for children. | Prison accommodation was built without children in mind hence lack of bedding facilities such as cot beds.  As the proportion of women in prisons grows so is the likelihood that the number of children either born or accompanying their mothers also grows. Governments in SSA cannot continue to ignore the presents of such innocent children behind the prison walls. |  |
|  | Zimbabwe  Children doing time with their mothers *-*. Retrieved from <http://www.irinnews.org/report/90137/zimbabwe-children-doing-time-their-mothers> Accessed 14 May 2018 | To explore experiences of mothers accompanied by their children in prisons, prison staff and NGO  perceptions | Zimbabwe | Qualitative. Interview with a female inmate, prison stuff and NGO representative | Zimbabwean jails have not escaped the country's decade-long economic malaise and food is in short supply.  A prison official who declined to be identified told IRIN that diseases such as tuberculosis and kwashiorkor, which affects mainly children and is caused by severe protein malnutrition, and pellagra, a vitamin deficiency disease, were prevalent.  A mother told IRIN that the three months of incarceration were taking their toll on her and the child.  Most of the children who died in prison were given pauper burials, either because their next of kin was not known, or families did not offer to pay for their burial.  A representative of the [Zimbabwe Network of People Living with AIDS](http://archive.kubatana.net/html/sectors/zim033.asp?sector=PRISON&year=2010&range_start=1) (ZNNP+), told IRIN that AIDS activists viewed the plight of children jailed with their mothers as a "serious problem" because their living conditions made it difficult for those who were HIV positive to obtain treatment.  The representative expressed extreme concern because it is a known fact that some of the children are born in prison to [HIV-] positive mothers and they also have the virus, while others were reportedly accompanying their mothers in prisons already sick. | Diseases in public health such as TB thrive in unhygienic and overcrowded environments. Alternative strategies should be explored to ensure the welfare and security of the needs of innocent children other than placement in prisons. |  |
|  | Zimbabwe: The untold stories of pregnant inmates Shout Africa. 2015. Available from: <http://www.shout-africa.com/news/zimbabwe-the-untold-stories-of-pregnant-inmates/> Accessed 26 May | To record experiences of ex-pregnant female prisoners | Zimbabwe | Qualitative  Interviews with 3 ex-prisoners, Deputy Commissioner of prisons an NGO representative working with prisons and a church organization | Harsh prison conditions with incidences of poor levels of sanitation, overcrowding and inadequate medical facilities.  Lack of water and clothing due to lack of inadequate funding from the fiscus  Toiletries used by inmates are provided by non-governmental and religious organizations. | Newly born babies are susceptible to hypothermia and the one extra blanket given immediate post-partum is inadequate. Resource gaps in prisons are present especially for children who are not included in the government budget. |  |
|  | Protecting Rights of Female Prisoners, Circumstantial Children Important, Times of Zambia Retrieved from  [http://www.times.co.zm](http://www.times.co.zm/?p=7357)  Accessed 14 May 2018. | To explore living conditions and needs of prisoners and circumstantial children in Zambian prisons | Zambia | Qualitative interviews with Lusaka Province Regional Commander, and consortium of three non-governmental organizations | 412 circumstantial children from the age of breastfeeding infants to five incarcerated with their mothers in 2011.  Sleeping conditions at Lusaka Central Prison do not provide incarcerated children with space that is safe and secure.  Observations made at Kamfinsa, Kansenshi and Mukobeko prisons, revealed that prisons are not a safe place for pregnant women, babies and young children.  There were no facilities for breastfeeding and expectant mothers. Incarcerated women with no alternative for childcare chose to have their infant and children under the age of four with them in prison.  Mothers have to share their meager food ration with their children in an environment lacking appropriate medical care that often-exposed children to disease. | Zambian prisons are characterised by poor diet, overcrowding and unhygienic conditions including lack of security for children. These poor conditions also predispose children to diseases that are detrimental to the children’s development |  |
|  | Prison Babies. When children are forced to live in jail. Daily Monitor  <https://www.monitor.co.ug/artsculture/Reviews/Prison-babies--When-children-are-forced-to-live-in-jail/-/691232/2012308/-/item/0/-/12jrldxz/-/index.html> Accessed 14 May 2018. | To Commemorate the Inter-Religious Council day to pray, eat and interact with inmates every year, prior to September 22, a UN marked day for peace celebrations were held in Moroto | Uganda | Visit to Moroto Prison, observations and interviews with at least two mothers in prison with their children the officer in charge, interreligious council representative and regional prisons commander | A female serving her sentence told the reporter that feeding and living in a crowded place with poor sanitation, with a child is what worried her.  “Sometimes our babies go without food. They suckle from morning to evening.  On the day of the visit one of the prison officers highlighted that the majority of children were dependent on their mothers’ milk and the prison was overwhelmed the prison was getting help from NGOs that supplemented the children’s feeding  Challenge shared was that school-going children did not have access to a day care centre unlike in Luzira and spent time in the prison cells with their mothers. | To ensure adequate milk for the growing child, breast feeding mothers need adequate nourishment. In the case of Uganda, it has been reported elsewhere that the government does not provide adequate funding for the upkeep of the children hence the overreliance on philanthropic organizations |  |
|  | Women with children in correctional centres: Parliamentary Monitoring Group (PMG). Gauteng Department of Correctional Services briefing https://pmg.org.za/committee-meeting/16961/  Accessed 14 Dec 2018 | To assess the position of women imprisoned with their babies and give feedback to the Committee to deal with concerns raised after the Committee’s visit to Pollsmoor Correctional Centre | South Africa Gauteng Department of Correctional Services | Visit to Pollsmoor Correctional Centre and feedback presentation | Presentation focused on the position of mothers with their babies either born in the correctional centre, or who needed to be with their mothers up to the age of two years, in terms of the Correctional Services Amendment Act  Concern to the DCS was that the facilities were not really meant to cater for babies.  Sixteen Female Correctional Centres had been designed with the Mother and Baby units to accommodate children in 22 correctional facilities as of December 2014  By the end of 2013 there were 87 babies with their mothers in Department of Correctional Services DCS facilities  DCS worked with other stakeholders, that include other government departments and NGOs, in the provision of health care services for the prevention of communicable and non-communicable conditions, pregnancy and post-partum care, including referral to specialists, and general promotion of health for women and their babies  Prevention of mother-to-child-HIV transmission, services were available, special diets for nursing mothers and children including vaccinations and health education.  Social work services and psychological services were also available upon request or referrals after proper assessments.  Registration of Mother and Baby units, as Early Childhood Development Centres, with the Department of Social Development (DSD) was a challenge because DCS facilities did not comply with the DSD’s strict norms and standards. Most of these structures were not designed to cater for the needs of babies. | The provision of mother baby units that is on the increase and comprehensive package of medical care should be models that other SSA governments can learn from. |  |
| **Academic Thesis** | | | | | | | |
| Twea, S. | Women as offenders – the social and legal circumstances of women who commit crimes: A case study of selected prisons in Malawi. Master Degree in Law. University of Zimbabwe. 2013.  http://hdl.handle.net/10646/1048. Accessed 20 April 2018. | To assess the social and legal implications of women who commit crime | Malawi | Qualitative  Interviews with 29 administrative staff at different levels and 30 women prisoners  Document review | Children were being maintained on adult diets except in rare circumstances when soy flour could be provided for children.  Overcrowding in narrow, poorly ventilated dark cells and sleeping difficult  Low standards of hygiene.  Some prisoners have serious health problems such as TB/HIV.  There were also indications that children were not adequately accessing under five clinics and basic immunization against polio, TB, diphtheria and measles. | Living conditions within prison settings endangers the health of children. Poor nutrition and unbalanced diet contribute to disease among children in this environment |  |
| Kamau NJ | Access to health care by inmates in Kenya: a study of Lang’ at a women’s prison and Nairobi remand/allocation. Master Degree Medical Sociology Thesis, University of Nairobi. 2006. [Available from: http://erepository.uonbi.ac.ke:8080/xmlui/handle/11295/17516  Accessed 24 May 2018 | To assess access to health care by inmates in Kenya: A study of Lang’ata women ‘s prison and Nairobi Remand Allocation | Kenya | Quantitative and qualitative  The questionnaires were administered to 30 males and 29 females  inmates and 8 key informants | There exists an elaborate system within which inmates’ access treatment while they are in prison or custody.  Barriers to access to medical care included bureaucracy and negative attitudes by medical personnel and prison warders.  Delays, long queues and pathetic conditions.  Lack of resources essential medicines, medical personnel and facilities  Female prisoners able to obtain essential medicines and treated by doctors and nurses.  Female prisoners also managed to get referrals to better health facilities, which had x-ray and laboratory facilities such as Kenyatta National Hospital  Majority of the inmates complained about the poor diet and its preparation, which they said led to their poor health and stomach problems.  It was the view of the voluntary health worker that the prisons lack water resulting to poor sanitary conditions. | Access to medical care, qualified personal and medicines was better for female in mates compared to their male counter parts but the care was not up to recommended international standards. Barriers to care included, bureaucracy and negative medical staff attitudes and prison guards |  |
| Malamba O | The Welfare of Children living with incarcerated mothers in selected prisons in Zambia. Master Thesis, The University of Zambia, 2016. <http://dspace.unza.zm:8080/xmlui/bitstream/handle/123456789/4874/Main>  Accessed 14 May 2018. | To ascertain the welfare of children living with incarcerated mothers in selected prisons of Zambia | Zambia | Qualitative reviewing books and documents;  25 respondents were selected 8 mothers from the Kabwe female prison and 4 mothers from the Lusaka Central Prison respectively. with; 5 prison wardens from each of the two prisons, making a total of 10 altogether, 2 prison officers in charge, one from Lusaka central and the other from Kabwe female prisons and the Director of Social Welfare Department and Focus Group Discussions were used on a group of prison wardens as well as a group of mothers living with their children in prison | 24 respondents out of 25 said that children were not given food in prison.  Children eat whatever the mother eats and that is samp or rice for breakfast, beans, kapenta and nshima for lunch or supper.  Prison authorities do not provide food for the children but instead only gives the mother because the Zambia prisons Act of 1966 does not include children in the dietary scale.  According to the Prisons Act, the dietary Scale is only for mothers and not their children.  There is no funding for circumstantial children hence the mother shares her share with her child. When the prisons were built, children were not catered for.  Mothers said foodgiven in prison was not balanced and clearly did not meet the dietary needs of children.  At both the Kabwe female prison and Lusaka Central prison, some well-wishers came to donate some items such as nappies and a (NGO) known as Prisons Care and Counselling Association (PRISCCA) donated items such as sugar, vaseline, baby powder and delight cereal porridge to the children.  All the mothers from Kabwe female prison said they were given same portions of food as non-breastfeeding mothers. However, a different view concerning food portions was given by one prison warder from Lusaka Central prison who said breast feeding mothers were given more food portions.  But the assertion was denied by the mothers  No separate accommodation for nursing mothers.  Children and mothers including other female inmates lived in 2 crammed cells at Kabwe while at Lusaka 2 of 4 mothers indicated there was no problem in sharing accommodation with other pregnant inmates  Sharing of accommodation was reported as a source worry for some nursing mothers who expressed concern of children getting infected as one pregnant woman who had diarrhoea while another pregnant woman had a terrible cough of which some mothers suspected to be Tuberculosis.  Some variations noted during the research included mothers in Lusaka central prison were only mixed with pregnant women and slept on bunk beds while their counter parts in Kabwe were mixed with other inmates and they slept on mattresses on the floor. | Variations in this study were observed. Poor diet for the mothers and their children are findings that have been reported in other SSA countries. Prison authorities by deliberating not putting children on a dietary scale also impacts on their ability to lobby for more resources for the upkeep of the children from the fiscus |  |
| Gowland I. | Moms Behind Bars: Motherhood in Eshowe Correctional Center" (2011). Independent Study Project (ISP) Collection. 1115. digitalcollections.sit.edu/cgi/viewcontent. Accessed 26 May 2018. | To explore the construction of motherhood within Eshowe Correctional Facility for Women. | South Africa | Qualitative sessions with a group of female inmates and informal interview with a mother and formal interview with three more people that included a social worker from Community Corrections, a division within the DCS, a social worker within the Department of Correctional Services, and a former inmate and current Phoenix facilitator | The Correctional Services Amendment Act of 2008, stipulates that incarcerated mothers in South Africa may keep their children in prison with them until the child reaches two years of age.  The Act also maintains that a special unit should exist where these mothers and children can live.  On August 18, 2011, the DCS opened the first model Mother and Child Unit attached to the Pollsmoor prison.  The new unit addresses the issues of deprivation for children imprisoned with their mothers and attempts to mitigate the negative effects of prison life.  The unit has a medical facility, a kitchen so mothers can prepare baby-friendly meals, a nursery, and an outdoor play area.  This was said to be the first unit of its kind in South Africa with plans afoot to establish three more facilities in the coming years  However as early as 2004, the ACHPR 2004 on its missions noted that a mother and baby unit in Durban existed | The Mother and Baby units that exist in South Africa are good practice that should be understudied by other governments within SSA and replicated |  |
| **United Nations Joint Programme on HIV/AIDS ( UNAIDS) United Nations Children's Fund (UNICEF ), United Nations Office of Drugs and Crime (UNODC), United Nations Political Office for Somalia (UNPOS) and Human Rights reports** | | | | | | | |
|  | United Nations Political Office for Somalia (UNPOS). Assessment of the Prison System in Mogadishu South. UNPOS/ UNICEF/ UNODC. 2012. <https://unpos.unmissions.org/unpos-and-human-rights> Accessed 24 May 2018. | To assess the prisons sector in South Central Somalia/Mogadishu. | Somalia | Inspection and qualitative interviews  Series of visits to the Mogadishu Central Prison and meetings with Government officials from various institutions within the criminal justice sector, including representatives from the Ministry of Justice, Religious Affairs and Endowment, Custodial Corps, and the Judiciary. Consultations with representatives of the Civil Society and interviews with prisoners detained at the Mogadishu Central Prison. | Children in prison with their mothers  Water shortage and no running water in the female wing  Medical care lacking  Shortage of equipment and staff  No soap  Lack of access to clean water and adequate sanitation facilities | The lack of basic amenities exposed infants, babies and children accompanying their mothers in prisons to infectious diseases some of which could be life-threatening |  |
|  | United Nations (UN) General Assembly. Report of the Special Rapporteur on torture and other cruel, inhuman or degrading treatment or punishment, Mission to Ghana. United Nations, New York. 2014 ww.ohchr.org/EN/HRBodies/HRC/RegularSessions/.../A-HRC-25-60-Add-1_en.doc  Accessed 28 Apr 2018. | To conduct an independent and objective scrutiny of Ghana’s human rights situation, particularly in regard to a number of critical issues in its criminal justice system and mental health-care practices. | Ghana | Meetings with  representatives of the Ministry of Foreign Affairs, the Ministry of the Interior, the Ministry of Justice and the Office of the Attorney–General, the chief psychiatrist of the Ministry of Health, members of the Ghana Police and Prisons Services, the Military Legal Directorate and the judiciary, and the Commissioner of the national human rights institution of Ghana, the Commission on Human Rights and Administrative Justice and representatives of United Nations agencies and non-governmental organizations (NGOs). | The Prisons Service has established a mother and baby unit at Nsawam prison, where women who give birth while in custody can keep their babies with them until the age of one. However, at Kumasi some women said their babies were removed after only a few months and then placed with a family member or social services. There is no additional budgetary allocation for supplementary food for pregnant or breastfeeding women. | Prisons lack of resources affects quantity and quality of food even for breast feeding mothers which in turn also affects quality and quantity of breast milk the babies get. Promising practices such as the mother and baby units should be upscaled to cover more correctional facilities |  |
| **Human Rights Organizations** | | | | | | | |
|  | Chad: Prisoners’ lives are threatened by appalling detention conditions. Amnesty International Canada. 2012.Available from: <https://www.amnesty.org/en/press-releases/2012/09/chad-prisoners-lives-are-threatened-appalling-detention-conditions-2012-09/> Accessed 27 May 2018 | To monitor human rights compliance | Chad | Observations in six prisons, document review, interviews with prisoners | Women and children held in overcrowded unhealthy environment  Nursing women, children or sick inmates such as those suffering from gastric problems or living with HIV/AIDS not given special diets  The lack of basic facilities and essential drugs complicated the work of the appointed nurses or health personnel.  No health workers or clinic at the newly opened prison.  Prisoners who were seriously ill were taken to N’Djamena general hospital, where family members have to buy medicine and pay the hospital bills.  Chadian prisons contribute to women’s ill-health and also exposes children, infants and babies living with their mothers in the prisons to infections which can be life-threatening | The children incarcerated with their mothers endure the same inhabitable prison conditions as their mothers. Inadequate medical provision and special diets also affects children. |  |
|  | The HIV and TB Prison Crisis in Southern Africa. Available from: <https://www.hrw.org/report/2010/04/27/unjust-and-unhealthy/hiv-tb-and-abuse-zambian-prisons> Accessed 31 May 2018. | To analyse prison health conditions in Zambia by independent human rights organizations. | Zambia | Qualitative interviews with 246 prisoners, eight former prisoners, 30 prison officers  Facility tours | Despite international standards calling for special provision for children incarcerated with their parents and the legal provision that, subject to the commissioner’s conditions, “the infant child of a woman prisoner may be received into the prison with its mother and may be supplied with clothing and necessaries at public expense,” and may stay up until age four, there is no food at all allocated to the children under age four who live with their mothers in prison facilities; they are expected to share out of the portion of the mother.  In situations where women are unable to breastfeed, the prison does not offer infant formula. Babies were reportedly sharing the poor diet with their children. The diet constituted of (beans and kapenta) and there was only one meal a day  Mothers had to make some porridge for children out of their nshima [a cornmeal porridge].  No budget allocated for the children  Mothers with their children under age four reported that children do not consistently receive adequate health care and face similar medical care challenges as incarcerated adults.  At Lusaka Central Prison a baby had died of diarrhoea was sick for three days before going to the clinic. | Despite the country having ratified international and regional protocols on child rights, the conditions under which young children are kept in Zambian prisons is a cause serious concern that should be addressed through monitoring by civil rights organizations. |  |
| Alison T. | Children living in prison: Insights from Sierra Leone. The Quaker United Nations Office, Geneva 2008.  advocaidsl.org/wp-content/uploads/2015/11/AdvocAid-Children-Living-in-Prison-Sierra-Leone.pdf  Accessed 9 Aug 2018. | To solicit the views of a wide range of stakeholders in order to ensure a balanced perspective of children living in prison. | Sierra Leone | Semi-structured interviews were carried out with women and mothers in Pademba Road Prison, released female prisoners who had been incarcerated with a child, prison officials and medical staff and other relevant government officials. | Open-air cells exposed to often flooding during the rainy season.  Mattresses and blankets are filthy and old, they are recycled between prisoners, and there are no regular provisions for new supplies.  Women and their children held in extremely cramped without adequate sanitary supplies or facilities, no toilets and inmates are forced to urinate and defecate in plastic buckets, that they must clean out and share also erratic water supplies  Most children are under the age of two and the availability of basic medical services as well as a sufficiently nutritious diet is of critical importance not only to their general development but also to their survival.  Most babies in prison are still breast-feeding.  Those weaned have access to the same food rations as are available to all prisoners, which usually consists of three small meals a day, most often consisting of rice and sauce.  Prison staffed by a full-time doctor and nurses who operate with extremely limited resources, both in terms of finances and medicine, and are stretched beyond capacity given that they have the health of over 1000 prisoners in their care.  Prison is rife with cases of malaria, typhoid and other communicable diseases  Medical personnel in the prison noted that the babies regularly fall ill and they attributed many of these health problems to the overcrowding, poor sanitation standards and malnourishment experienced inside prison walls  Prison does not provide baby clothing for imprisoned babies and no access to basic precautionary health measures such as mosquito nets or soap for personal use.  Prison doctor generally tends to enter the women’s section when specifically summoned by a prison officer regarding detainees’ ill health and, nurses tasked with monitoring on a daily basis, routine check-ups of prisoners and/or their babies in the prison are rarely conducted.  Supplies donated by charitable organisations were not going directly to mothers and their babies but are often taken and distributed by prison officers – with guards sometimes taking supplies for their own families or distributing goods to favoured inmates. | The prison guards help themselves to donations meant for mothers and their young children. Prison authorities must have checks and balances through intensive monitoring of staff to ensure that donated goods are distributed equitably to the target beneficiaries. |  |
| Sheehan F, Mukisa D. | A shared sentence: children of imprisoned parents in Uganda. A report on the implementation of General Comment No.1 (Article 30 of the African Charter on the Rights and Welfare of the Child)  Penal Reform International, London. 2015. https://cdn.penalreform.org/wp-content/uploads/2015/11/PRI_FHRI-Report_-Implementation-of-GC-No1-WEB.pd. Accessed 14 Apr 2018. | To assess how the General Comment has been implemented so far and highlighting some of the gaps that need to be filled. | Uganda | Qualitative drawing upon document and data review as well as interviews and focus group discussions with those engaged in working with children of imprisoned parents including lawyers and paralegals, NGOs, prison monitoring bodies, social workers, prison officers and the judiciary. Interviews with a small sample of 15 children of imprisoned parents and 11 mothers in prison | Children are allowed to stay with their mothers in prison up to the age of 18 months  The children can stay longer in the absence of other alternatives to care by extended family or NGO to take care of them.  Conditions were described as very poor with most basic necessities not available  Interviewed mothers complained of lack of food and inadequate hygiene in particular. Inadequate funding for these children as highlighted during an interview and children were not included in the government budget.  Provision for the children was through the good will of the civil society, well-wishers and also the innovativeness of the prisons officers’ | Poor resourcing of penal institutions impact on the care that mothers and their children get. Civic society should lobby the government to increase funding to these institutions |  |
| **African Union Reports** | | | | | | | |
|  | African Commission on Human and Peoples’ Rights (ACHPR). Malawi: Mission on Prisons and Conditions of Detention - 2001/Malawi/States/ACHPR. L'Exprimeur, Paris. 2002 http://www.achpr.org/states/malawi/missions/prisons-2001/. Accessed 20 April 2018. | To assess and document the conditions of detention in Malawi. | Malawi | Consultations, inspections and interviews 13 prisons visited, 4 consultations with authorities, 6 with NGOs, 5 with media houses and prisoners’ interviews in private, individually or in groups in each prison and police station visited. | The conditions of detention of women are more humane in Malawi but the mission reports that the same view could not be extended to the old women, expecting mothers and women detained with their children.  Prison is not a safe place for, babies and young children and it is not advisable to separate babies and young children from their mothers.  The Special Rapporteur strongly recommended that these women and children should be released as soon as possible so that they can be taken care of properly by the community. | Prisons are not a safe environment for young children to grow up in and releasing the mothers allows the children to grow in a safe secure environment. Alternative imprisonment could be considered such as community service for the mother offender |  |
|  | African Commission on Human and Peoples’ Rights (ACHPR). Mozambique: Mission on Prisons and Conditions of Detention - 2001/ Mozambique/ States/ACHPR. Gambia, 2001.http://www.achpr.org/states/mozambique/missions/prisons-2001/. Accessed 26 May 2018. | To assess the implementation of the recommendations made by the Report of the Special Rapporteur in 1997 | Mozambique | Inspections, interviews, meetings observations and document review. eight prisons visited and one police station, Consultations undertaken with prison authorities 16 including contracted physicians, nurses and helpers at places visited, NGOs eight, and prisoners | Some women were detained with their children who needed regular specific attention from specialist staff  Overcrowding  Inadequate centres and medical staff  Food and water/sanitation problems.  Hygiene problems due to lack of soap, of cleaning tools, and limited access to bathing facilities and water. | Lack of specialized regular medical care and inadequate medical staff compromised the health of the children. Given multiplicity of factors a coordinated approach to service availability and accessibility by different partners is warranted. |  |
|  | African Commission on Human and Peoples’ Rights (ACHPR). Namibia: Mission on Prisons and Conditions of Detention - 2001/Namibia/States/ACHPR. Niger, 2001. http://www.achpr.org/states/namibia/missions/prisons-2001/  Accessed 26 May 2018. | To assess and document the conditions of detention in Namibia. | Namibia | Interviews, Observations and document review of 10 prisons  Consultations with policy makers at various levels and administrators, various stakeholders including NGOs and the media, individual prisoners and of in groups | Food Menus and quantities are established by a nutritionist varying in principle according to gender, age and state of health.  Babies and infants detained with their mothers are supplied with a special diet.  Children detained with their mothers as well as the sick prisoners, may, on doctor's prescription, receive fresh fruit, more milk and eggs. | The inclusion of a nutritionist in the staff complement ensures that the quality and quality of food given to children is sufficient and the diet well balanced. This is a good practice that other SSA countries can adopt. |  |
|  | African Commission on Human and Peoples’ Rights (ACHPR). Uganda: Mission on Prisons and Conditions of Detention - 2001/Uganda/States/ACHPR. Niger. 2003. http://www.achpr.org/states/uganda/missions/prisons-2001/. Accessed 26 May 2018. | To evaluate and document conditions of detention in Uganda | Uganda | Interviews, meetings Observations and document review  13 prisons visited, 6 police stations, 1 remand home for juveniles, discussion conducted with 7 authorities3 NGOs representatives, 9 representatives of media houses and individual prisoners and in groups. | The women's cell at Kasese police station accommodated between five and seven prisoners at a time, for a 6 m2 surface.  The cell had no light and no toilet. Their ward in Tororo prison farm had no lights, and poor ventilation. It accommodated 12 women and one baby  In Masindi, there was a separated space for mothers with children  Even babies got poshow (maize meal) and beans, and porridge without sugar, no milk.  The three women with children did not receive any visit (and therefore no additional food) since they were too far away from their families.  One of them was ill and could not breast feed her child.  In Tororo Prison farm, one baby was staying with his mother. He got milk and sugar everyday  In Mbale women prison, the baby got cow milk, but no diapers nor clothes.  In Luzira women prison, there were 24 kids up to 3 years, and 9 pregnant women.  It was difficult to obtain a special diet for babies, but they did get milk, with the help of NGOs and the Red Cross. | Basic amenities lacking for children and affecting access to health care and service provision. The Justice system cannot address all these challenges on its own but need a coordinated approach that includes the private sector, local and international NGOs |  |
|  | African Commission on Human and Peoples’ Rights (ACHPR). Cameroon: Prisons and Detention Conditions - 2002/Cameroon/States/ACHPR. Gambia, 2002. http://www.achpr.org/states/cameroon/missions/prisons-2002/. Accessed 26 April 2018. | To evaluate and document conditions of detention in Cameroon. | Cameroon | Interviews, meetings observations and document review  8 prisons and 4 police stations visited. Consultations with 24 with authorities, 4 NGOs, 1research institutions and 8 media houses | Lack of basic amenities like soaps and toiletries, and the alarming rate of congestion.  There is a strong stench in the various premises. Police cells emit the worst odour (hygiene and toilets)  Sanitation materials are not regularly and adequately issued to inmates.  Water is highly scarce and there is a deplorable waste management.  The menu is poor and the quantity inadequate.  Some women are imprisoned with their children who eat the same food as adults, thereby potentially affecting their growth.  Food badly cooked, insufficient, and lacking good taste.  Lack of medical staff. | Conditions impacting on health service provision to women prisoners and their children are similar to other mission reports and need a holistic approach to addressing them |  |
|  | African Commission on Human and Peoples’ Rights (ACHPR). Ethiopia: Mission on Prisons and Conditions of Detention - 2004/Ethiopia/States/ACHPR. Gambia, 2004. <http://www.achpr.org/states/ethiopia/missions/prisons-2004/>  Accessed 26 April 2018 | To monitor prisons and other places of detention in Member States of the African Union | Ethiopia | Qualitative meetings, observations and document review  visited and inspected nine prisons, two prison farms and two police stations. | There is a separate section for nursing mothers and expectant mothers.  The toilets are not very clean and the section for nursing mothers a bit untidy.  Expectant mothers, nursing mothers, children and sick prisoners are given special meals.  According to Ethiopian prison regulations, these children are supposed to remain with their mothers until they reach the age of 18 months.  Children remain in prison with their mothers for as long as their mothers are detained or they (the children) are able to fend for themselves.  There are children in prison aged up to 8 years. Most of the children are not going to school and have no toys or other entertaining facilities for children.  Other basic items such as soap, sheets, and detergents not provided. | Although a shortage of health facilities and other necessities was reported the prison was able to provide special meals for nursing mothers, the sick and children. Children remaining in prisons beyond the age of five years had no access to education which is violation to the child’s right to education. |  |
|  | African Commission on Human and Peoples’ Rights (ACHPR). South Africa: Prisons and Detention Conditions - 2004/South Africa/States/ACHPR. Gambia, 2004. http://www.achpr.org/states/south-africa/missions/prisons-2004/. Accessed 26 May 2018. | To draw the attention of prison officials to the treatment of persons deprived of their liberty. | South Africa | Qualitative and quantitative Inspections, interviews, meetings with national and local prison officials, closed door meetings with detainees, and communications from civil society organisations and information from various organisations i.e. 19 Detention centres, one mental hospital, one, repatriation centres, six prisons, one Child Justice Centre, Prisons six, Police stations four, youth centre one and eight institutions | Mother child Unit in Durban and the children are provided with special diet.  The female section has a unit for nursing mothers called the Mother-Child Unit.  The mother and child unit is in a separate section within the Centre and holds only prisoners with babies.  The children are provided with special diet – milk and fruits.  The mothers are given two soaps a month for themselves and the babies.  They however complained that the food given to the children was not enough.  They need extra nappies for the children.  According to the inmates, the authorities allowed only the following items to be given to the kids – chocolate milk, ordinary milk, noodles, baby juice, cerelac and maltivite.  They argue that these items are for children of one year and under but there are children who are more than one year to three years who need more than just the items provided by the authorities.  In terms of South African law, children can stay with their mothers in prison for up to 2 years and later can be taken by their families or transferred to foster parents.  Age ranges of children were from 1 year to 5 years  All the prisons visited, had an in-house clinic that provides first aid treatment for minor illnesses. In house clinics lacked l laboratory equipment or have very few medicines in the dispensaries (lack of medication).  Provision of basic items like soap, and detergents for washing clothes. | Children had access to better nutrition and medical care in comparison to most children accompanying their mothers in prisons in most of the SSA countries. This is good practice that should be replicated and scaled up across SSA prisons for the benefit of not only these unfortunate children who are innocent but find themselves serving prison terms of their mothers for crimes they have not committed. |  |
|  | African Commission on Human and Peoples’ Rights (ACHPR). Report of the Special Rapporteur on Prisons and Conditions of Detention in Africa. 52nd Ordinary Session, African Commission on Human and Peoples’ Rights. Yamoussoukro, Côte d’Ivoire. 2012. http://www.achpr.org/sessions/52nd/. Accessed 23 May 2018. | To assess progress made addressing and implementing recommendations made to different countries taken by the mission and also challenges impacting on implementation | All countries in Africa and including SSA | Presentations, meetings and plenary discussions | With regard to women, the Protocol to the African Charter on the Right of women in Africa guarantees the right to integrity and security of women, pregnant and nursing mothers in detention, prohibits sexual violence in private and public and imposition of death penalty on pregnant and nursing mothers.  There are several critical problems faced by women in prison - most are unmet in the prison environment.  Women in prison have experienced victimization, unstable family life, school and work failure, and substance abuse and mental health problems.  Women prisoners are particularly vulnerable to sexual abuse by prison guards whether in female or mixed prisons  Prisons are not a safe place for pregnant women, babies and young children and it is not advisable to separate babies and young children from their mothers.  Lack of sufficient treatment facilities is increasing the risk of mother-to-child HIV transmission (PMTCT), HIV prevention treatment care and support | A rights-based approach to interventions in prisons have been implemented but conditions in Africa including SSA remain poor with women and children mostly affected. Lack of sufficient treatment facilities for PMTCT increases vertical transmission of HIV to the unborn baby |  |
| **United States Department of State Bureau of Democracy, Human Rights and Labor 2017** | | | | | | | |
|  | United States Department of State. Bureau of Democracy, Human Rights and Labor. Senegal 2016. Human Rights Report. Washington, D.C. 2017. https://www.state.gov/documents/organization/277283.pdf. Accessed 19 April 2018. | To report on human rights practices and situation in the country | Senegal | Desk review of reports by independent monitoring visits of local human rights groups, international observers and ICRC | Harsh prison and dentition centre conditions potentially life threatening attributed to food shortages, overcrowding, poor sanitation and inadequate medical care.  Infants and new-borns were often kept in prison with their mothers until age one, with no special cells, additional medical provisions, or extra food rations.  The National Organization for Human Rights, a nongovernmental organization (NGO), also identified lack of adequate sanitation as a major problem. Poor and insufficient food, stifling heat, poor ventilation, poor drainage, and insect infestations also were problems throughout the prison system  Food shortages and poor in both quantity and quality  Inadequate medical care with access controlled by prison guards | Common thread running through the countries are harsh penal conditions under which mothers and their children are held that range from overcrowding, poor sanitation poor quality and quantity of food to inadequate medical care. |  |
|  | United States Department of State Bureau of Democracy, Human Rights and Labor. Cote D’ivoire 2017 Human Rights Report. Washington, D.C. 2017. https://www.state.gov/documents/organization/277235.pdf. Accessed 19 April 2018. | To report on human rights practices and situation in the country | Cote D’ivoire | Desk review of independent monitoring reports through visits by United Nations and local and international NGOs | Prison conditions poor and life-threatening  Overcrowding with inadequate sanitary conditions, worsened by lack of portable water  Poorly trained staff and lack of medical care.  Inadequate food  Philanthropic organizations sometimes financed prisoners’ medical care.  The children often lived with their mothers in prison and prisons accepted no responsibility for their care or feeding.  Inmate mothers received help from local and international NGOs. |  |  |
|  | United States Department of State Bureau of Democracy, Human Rights and Labor. Madagascar 2017 Human Rights Report. Washington, D.C. 2017. https://www.state.gov/documents/organization/277261.pdf. Accessed 19 April 2018. | To report on human rights practices and situation in the country | Madagascar | Desk review of independent monitoring reports through visits by NGOs, diplomats, and international human rights observers and the International Committee of the Red Cross (ICRC) | Deteriorating prison infrastructure that often-lacked sanitation facilities and potable water were reported  Some under school age children shared cells with their incarcerated mothers.  Disease as a result of poor hygiene resulted and infestations of insects and rodents.  Conditions in prisons described as poor with overcrowding  Limited access to potable water, inadequate sanitation, insufficient ventilation and lighting, overcrowding  Inadequate medical facilities. |  |  |
|  | United States Department of State Bureau of Democracy, Human Rights and Labor. Djibouti 2017 human rights report. Washington, D.C. 2017. https://www.state.gov/documents/organization/277237.pdf. Accessed 16 April 2018. | To report on human rights practices and situation in the country | Djibouti | Desk review of independent monitoring reports through visits by United Nations and local and international NGOs | Poor lighting and heating, limited potable water, ventilation, and inhuman sanitation conditions  Gabode Prison conditions of detention for women were similar to those for men, although less crowded  Authorities allowed young children of female prisoners to stay with their mothers. | Although less crowding was reported, poor detention conditions were also observed |  |
|  | Inventory of countries that allow mothers to stay with their children in penal institutions.  Global Research Directorate. Library of Congress. Laws on Children Residing with Parents in Prison. Washington, DC. 2014. https://www.loc.gov/law/help/children-residing-with-parents-in-prison/foreign.php. Accessed 4 Sep 2018. | To conduct an inventory of Laws that allow mothers to stay with their children in prison in the country | Benin | Review of country laws and policies on children | The law reported as allowing mothers to keep their young children with them in prison although no special accommodation seems to be provided for such situations. | Despite the law allowing mothers to stay in prison with their children no separate accommodation was for nursing mothers was provided. |  |
|  |  | To conduct an inventory of Laws that allow mothers to stay with their children in prison in the country | Burundi | Review of country laws and policies on children | The law appears to allow imprisoned mothers to have their children live with them  No special accommodation is provided for these children and their incarcerated parent, and only nine of them had access to special health services and clothing provided by a charity organization |  |  |
|  |  | To conduct an inventory of Laws that allow mothers to stay with their children in prison in the country | Cameroon | Review of country laws and policies on children | The law allows housing young children who were born there to incarcerated mothers  No special accommodation seems to exist for these children and their mothers, and the Yaoundé central prison is extremely overpopulated |  |  |
|  |  | To conduct an inventory of Laws that allow mothers to stay with their children in prison in the country | Côte d’Ivoire | Review of country laws and policies on children | Ivorian law allows children to stay in prison with their incarcerated mothers  No separate accommodation for nursing mothers and their children |  |  |
|  |  | To conduct an inventory of Laws that allow mothers to stay with their children in prison in the country | Botswana | Review of country laws and policies on children | The law allows breastfeeding children of female prisoners to live with the mother until the child has been weaned.  During the child’s stay in prison, the child may be provided with “clothing and necessaries at the public expense | Although statutes exist and most leaders have ratified international and regional protocols, there is a variance between the law, policy and practice |  |
|  |  | To conduct an inventory of Laws that allow mothers to stay with their children in prison in the country | Ethiopia | Review of country laws and policies on children | Children under the age of 18 months can stay with their incarcerated mothers  The Federal Prison Administration (the Administration) is required to provide the infant with “the necessary food, vaccination, medical care as well as other necessary items.” |  |  |
|  |  | To conduct an inventory of Laws that allow mothers to stay with their children in prison in the country | Kenya | Review of country laws and policies on children | The law allows infant children of female prisoners to stay with their mothers in certain circumstances  The law states that: “the infant child of a female prisoner may be received into prison with its mother and may be supplied with clothing and necessaries at public expense” until the age of four years or other alternative sources of care can be found |  |  |
|  |  | To conduct an inventory of Laws that allow mothers to stay with their children in prison in the country | Malawi | Review of country laws and policies on children | Malawian law provides that a breastfeeding child of a female prisoner may be permitted to live with the mother until the child has been weaned.  During the child’s stay with the mother, the child may be provided with “clothing and necessaries at the public expense |  |  |
|  |  | To conduct an inventory of Laws that allow mothers to stay with their children in prison in the country | Swaziland | Review of country laws and policies on children | The law allows breastfeeding children of female prisoners to live with the mothers until the child is weaned, during which time the child may be provided with “clothing and necessaries” at the public expense |  |  |
|  |  | To conduct an inventory of Laws that allow mothers to stay with their children in prison in the country | Namibia | Review of country laws and policies on children | The law permits female prisoners with children to temporarily bring the infant children to reside with them in correctional facilities  The law mandates that the state provide such a child “clothing and other necessaries” for as long as the child remains in the correctional facility. |  |  |
|  |  | To conduct an inventory of Laws that allow mothers to stay with their children in prison in the country | Uganda | Review of country laws and policies on children | Ugandan law permits children under the age of eighteen months to join their mothers in prisons until the age of eighteen months  The law provides for the provision of “special facilities needed for their condition.”  The law also mandates that the state provide clothing and other “necessities of life” to infants living with their mothers in prison until they reach the age when they are to be removed from the prison |  |  |
|  |  | To conduct an inventory of Laws that allow mothers to stay with their children in prison in the country | Zambia | Review of country laws and policies on children | The law permits an infant child under the age of four of a woman prisoner to live with the mother  An HRW report stated that “[the] Prison Service did not have special diets for children who go in prison with their mothers at the time of the visits. Inmate mothers shared their food rations with their children and clothing, bathing or washing soaps were not provided for these children. |  |  |
|  |  | To conduct an inventory of Laws that allow mothers to stay with their children in prison in the country | South Africa | Review of country laws and policies on children | The law allowed mothers to stay with their children in correctional facilities up to the age of 2 years  The Department of Correctional services whenever possible, is required to ensure the availability of “a mother and child unit” to accommodate inmates with children  While the child is in its custody, the Department is responsible for the child’s food, clothing, health care, and other facilities necessary for the child’s “sound development” | Although South Africa might have challenges in some areas of their correctional facilities in SSA, there are also some good prison practices that other countries could learn from. |  |
|  |  | To conduct an inventory of Laws that allow mothers to stay with their children in prison in the country | Nigeria | Review of country laws and policies on children | The federal law provides that a child of a female prisoner may be allowed to reside with the mother if the child is breastfeeding and less than eighteen months old | While the law allowed mothers to keep their babies in prison it is silent on the provision of basic needs such as clothing health care and food for the children |  |
|  |  | To conduct an inventory of Laws that allow mothers to stay with their children in prison in the country | Sierra Leone | Review of country laws and policies on children | Data from secondary sources indicated that the law allowed breastfeeding mothers to stay with their children during incarceration up the age of two years |  |  |
|  |  | To conduct an inventory of Laws that allow mothers to stay with their children in prison in the country | South Sudan | Review of country laws and policies on children | South Sudan law provides that a child of a female prisoner may stay with the mother in prison until the child is two years of age |  |  |
|  |  | To conduct an inventory of Laws that allow mothers to stay with their children in prison in the country | Zimbabwe | Review of country laws and policies on children | A female prisoner in Zimbabwe may be allowed to bring her breastfeeding child to live with her in prison until the child is weaned |  |  |
|  |  | To conduct an inventory of Laws that allow mothers to stay with their children in prison in the country | Tanzania | Review of country laws and policies on children | The Tanzanian (mainland) law provides allows an infant child of a female prisoner to reside in prison with his/her mother  Children staying in prisons with their mothers live in poor conditions with no special diet, and are thus forced to share food with their mothers | Overcrowding and poor quality and quantity of food compromises the health and developmental milestones of the children |  |
